# Opposing effects on cardiac function by calorie restriction in different‐aged mice

**DOI:** 10.1111/acel.12652

**Published:** 2017-08-11

**Authors:** Yunlu Sheng, Shan Lv, Min Huang, Yifan Lv, Jing Yu, Juan Liu, Tingting Tang, Hanmei Qi, Wenjuan Di, Guoxian Ding

**Affiliations:** ^1^ Department of Geratology The First Hospital Affiliated to Nanjing Medical University 300 Guangzhou Road Nanjing 210029 China

**Keywords:** AMPK, autophagy, calorie restriction, cardiac aging, FOXO

## Abstract

Calorie restriction (CR) increases average and maximum lifespan and exhibits an apparent beneficial impact on age‐related diseases. Several studies have shown that CR initiated either in middle or old age could improve ischemic tolerance and rejuvenate the aging heart; however, the data are not uniform when initiated in young. The accurate time to initiate CR providing maximum benefits for cardiac remodeling and function during aging remains unclear. Thus, whether a similar degree of CR initiated in mice of different ages could exert a similar effect on myocardial protection was investigated in this study. C57BL/6 mice were subjected to a calorically restricted diet (40% less than the *ad libitum* diet) for 3 months initiated in 3, 12, and 19 months. It was found that CR significantly reversed the aging phenotypes of middle‐aged and old mice including cardiac remodeling (cardiomyocyte hypertrophy and cardiac fibrosis), inflammation, mitochondrial damage, telomere shortening, as well as senescence‐associated markers but accelerated in young mice. Furthermore, whole‐genome microarray demonstrated that the AMP‐activated protein kinase (AMPK)–Forkhead box subgroup ‘O’ (FOXO) pathway might be a major contributor to contrasting regulation by CR initiated in different ages; thus, increased autophagy was seen in middle‐aged and old mice but decreased in young mice. Together, the findings demonstrated promising myocardial protection by 40% CR should be initiated in middle or old age that may have vital implications for the practical nutritional regimen.

## Introduction

CR is defined as a 20–50% reduction in calorie intake than *ad libitum* (AL) level without malnutrition. Over the last several decades, CR has been known to prolong average and maximum lifespan in a wide variety of organisms (Finkel, 2015). In addition, hitherto, CR is the most successful intervention for delaying aging progression or the development of age‐related chronic diseases (Speakman & Mitchell, 2011). Cardiac aging is a complex pathophysiological process accompanied by a number of biological events including cardiac remodeling and dysfunction (Boengler *et al*., 2009). Although CR was found to be highly effective in lowering blood pressure, decreasing systemic inflammation, and improving cardiac diastolic parameters (Fontana *et al*., 2004; Fontana, 2008), almost all previous reports were focused on pathological states such as obesity and myocardial ischemia. Whether the anti‐aging effects of CR also extend to cardiac functions in physiological conditions is currently unknown.

Moreover, several studies showed that CR initiated either in middle or old age could improve ischemic tolerance and rejuvenate the aging heart (Peart *et al*., 2012; Yan *et al*., 2013; Dai *et al*., 2014); however, the data are not uniform when CR initiated in young age. For example, some studies have indicated that CR initiated in young could improve ischemic tolerance and impaired cardiac structure and function of subjects with metabolic disorders (Viljanen *et al*., 2009; Sung *et al*., 2011; Noyan *et al*., 2015). Contrastingly, the benefits of CR on mitochondria and cardiac function could not be observed in the young healthy heart (Hancock *et al*., 2011). Thus, the exact time when CR initiation is most beneficial for cardiac remodeling and function during aging yet remains unclear.

Additionally, the degree of CR is usually 20–50% less than AL level, which exerts a maximum beneficial impact on aging and lifespan, although some evidence suggests that CR can have harmful effects when the degree is extremely stringent. A previous study reported that a 3‐day partial (471 kcal day^−1^) and a 3‐day complete starvation (normal diet intake 2065 kcal day^−1^) induced a dose‐dependent increase in myocardial triglyceride (TG) content and a decrease in diastolic function in healthy males (Hammer *et al*., 2008). Another research published in diabetes also showed that a very low‐calorie diet (471 kcal day^−1^) for 3 days decreased left ventricular diastolic function (van der Meer *et al*., 2007).

Taken together, in the present study, we investigated whether a similar degree of CR could have a similar myocardial protection in mice during aging. The C57BL/6 mice were fed on a calorically restricted diet (40% less than the AL diet, the convinced beneficial degree of CR) for 3 months initiated at different ages (3, 12, and 19 months), and cardiac remodeling, cardiac function, and aging phenotypes were assessed.

Furthermore, CR modifies several critical pathways, such as the insulin growth factor‐1 (IGF‐1) receptor system, mammalian target of rapamycin (mTOR), and regulation of the respiratory metabolism such as AMPK and sirtuins, in the organisms (Lopez‐Otin *et al*., 2016). However, the underlying mechanisms of the cardiac effect of CR in differently aged mice have not been previously investigated. Thus, we performed whole‐genome and miRNA microarray in myocardial tissue to explore the potential mechanisms of the regulation of cardiac physiology by CR in different‐aged mice. Together, we aimed to propose an effective nutritional regimen that may be promising in cardiac protection during aging.

## Results

### Overview of aging‐ and CR‐induced effects on cardiac function

As shown in Table 1, body weight was increased both in middle‐aged (15 months‐AL) and old (22 months‐AL) mice compared to young mice (6 months‐AL). Heart weight and heart weight/tibia length were increased in old mice when compared to both young and middle‐aged mice; there was only an upward trend in middle‐aged mice when compared to young mice. All these three parameters showed a decrease after CR at three different ages. Echocardiography showed impaired cardiac systolic function with aging as evidenced by decreased ejection fraction (EF) and fractional shortening (FS) (Table 1, Fig. S1, Supporting information). Then, we investigated the effect of CR initiated in different ages on cardiac contractile function. In response to CR, EF and FS that decreased with aging were significantly improved in both middle‐aged and old mice (FS in old mice, *P = *0.06, Table 1, Fig. S1, Supporting information). Conversely, EF and FS significantly decreased after CR in young mice, suggesting that in healthy young mice, CR induced cardiac systolic dysfunction (Table 1, Fig. S1, Supporting information).

**Table 1:** General biometric and echocardiographic characteristics in AL and CR mice at 6, 15, and 22 months of age

Parameters	6 months		15 months		22 months	
	AL	CR	AL	CR	AL	CR
Body Weight (g)	26.86 ± 2.04	21.08 ± 2.12**	34.82 ± 3.22##	27.21 ± 3.24**	33.31 ± 2.82##	27.68 ± 2.63**
Heart Weight (mg)	119.91 ± 8.73	94.97 ± 15.07**	135.33 ± 12.44	117.19 ± 9.78*	157.16 ± 14.32## ^,^ &&	140.88 ± 7.18**
HW/TL (mg mm^−1^)	6.46 ± 0.44	5.18 ± 0.81**	7.19 ± 0.74	6.15 ± 0.55**	8.73 ± 0.79## ^,^ &&	7.82 ± 0.39**
LVEDD (mm)	3.46 ± 0.35	3.32 ± 0.38	3.89 ± 0.44	3.91 ± 0.29	3.56 ± 0.44	3.74 ± 0.19
LVESD (mm)	2.33 ± 0.4	2.49 ± 0.17	2.89 ± 0.4	2.65 ± 0.27	2.85 ± 0.67	2.63 ± 0.26
IVSD (mm)	1.08 ± 0.03	0.89 ± 0.07**	0.99 ± 0.11	0.96 ± 0.12	1.16 ± 0.13	0.94 ± 0.07**
IVSS (mm)	1.6 ± 0.12	1.18 ± 0.14**	1.37 ± 0.22	1.53 ± 0.16	1.50 ± 0.25	1.47 ± 0.11
PWD (mm)	0.65 ± 0.21	0.7 ± 0.11	0.72 ± 0.09	0.7 ± 0.08	0.85 ± 0.28	0.9 ± 0.1
PWS (mm)	1.01 ± 0.35	0.96 ± 0.11	0.89 ± 0.17	1.01 ± 0.2	0.97 ± 0.19	1.21 ± 0.19
EF (%)	62.53 ± 7.18	49.78 ± 7.18**	50.82 ± 4.76##	58.86 ± 3.68*	45.63 ± 8.62##	56.79 ± 6.69*
FS (%)	33.12 ± 4.91	24.63 ± 4.42**	25.66 ± 3.06##	30.68 ± 2.41*	22.44 ± 4.93##	29.29 ± 4.35
LV Mass (mg)	103.86 ± 9.4	86.48 ± 5.51	145.19 ± 21.98##	109.99 ± 10.41**	148.89 ± 19.67##	114.57 ± 13.69*
LV Mass Corrected (mg)	83.09 ± 4.82	69.18 ± 6.71	116.16 ± 17.58##	87.99 ± 8.33**	119.11 ± 15.73##	91.66 ± 10.95*
HR (beats per min)	360 ± 17	391 ± 31	458 ± 20##	432 ± 58	475 ± 22##	452 ± 30

Data are mean ± SEM (*n* = 6–10 mice/group).

HW/TL, heart weight/tibia length; LVEDD, left ventricular end‐diastolic dimension; LVESD, left ventricular end‐systolic dimension; IVSD, septum thickness in diastole; IVSS, septum thickness in systole; PWD, left ventricular posterior wall in diastole; PWS, left ventricular posterior wall in systole; EF, ejection fraction; FS, fractional shortening; HR, heart rate.

*P < 0.05 vs. age‐matched AL group, **P < 0.01 vs. age‐matched AL group. #*P* < 0.05 vs. 6 months (6 m) AL group, ##*P* < 0.01 vs. 6 months AL group. &*P* < 0.05 vs. 15 months (15 m) AL group, &&*P* < 0.01 vs. 15 months AL group.

### Effects of 3 months CR initiated in different ages on cardiomyocyte hypertrophy

**Figure 1:**
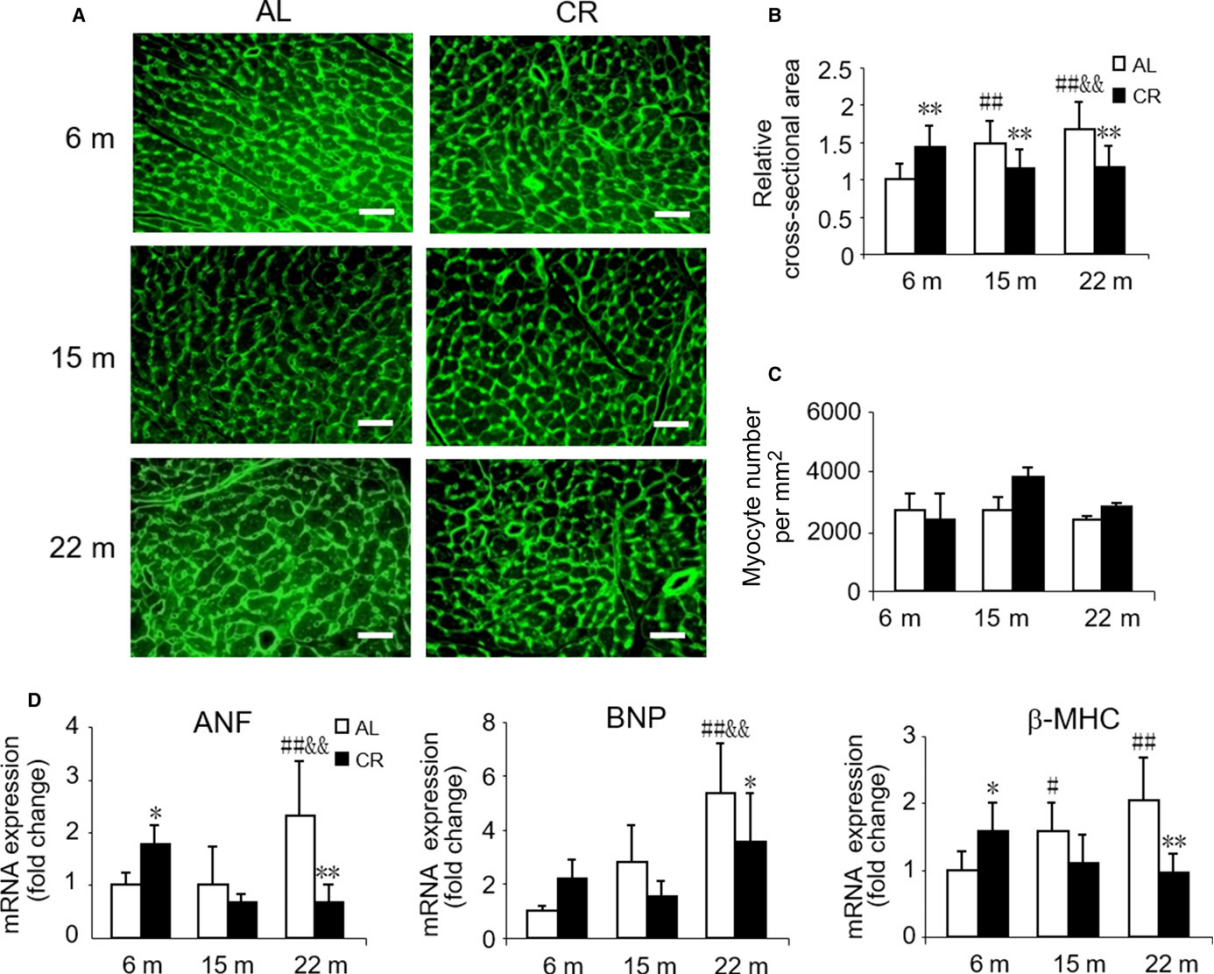
Effect of age and CR on cardiomyocyte hypertrophy. (A) Representative images using FITC‐conjugated WGA staining for cardiac tissues from AL and CR mice at 6, 15, and 22 months of age. Scale bars, 20 μm. (B) Quantitation of cardiomyocyte cross‐sectional area. Mean ± SEM,* n* = 200 cardiomyocytes for cross‐sectional area quantification. (C) Quantitation of cardiomyocyte number. Mean ± SEM,* n* = 3 whole cross sections of left ventricles per group for cardiomyocyte number quantification. (D) mRNA quantification of the hypertrophic marker genes. *P < 0.05 vs. age‐matched AL group, **P < 0.01 vs. age‐matched AL group. #*P* < 0.05 vs. 6 months AL group, ##*P* < 0.01 vs. 6 months AL group. &*P* < 0.05 vs. 15 months AL group, &&*P* < 0.01 vs. 15 months AL group.

Consistent with the increased heart weight, the cross‐sectional area (CSA) of cardiomyocytes increased with aging as observed by the wheat germ agglutinin (WGA) staining (Fig. 1A,B). Moreover, the mRNA levels of hypertrophic markers, atrial natriuretic factor (*ANF*), and brain natriuretic peptide (*BNP*) were greater in old mice vs. other ages, and β*‐MHC*, another hypertrophic marker, the mRNA level of which was higher in both middle‐aged and old mice vs. young mice (Fig. 1D). In response to CR, the CSA of cardiomyocytes significantly decreased in middle‐aged and old mice but increased in young mice (Fig. 1A,B). In addition, the number of cardiomyocytes were predisposed to increase as a result of CR in middle‐aged and old mice but were inclined to decrease in young mice (Fig. 1C). Furthermore, the expression of *ANF*,* BNP*, and β*‐MHC* was also downregulated by CR in old mice while it upregulated in young mice, and no significant difference was observed by CR in middle‐aged mice (Fig. 1D).

### Effects of 3 months CR initiated in different ages on cardiac fibrosis

**Figure 2:**
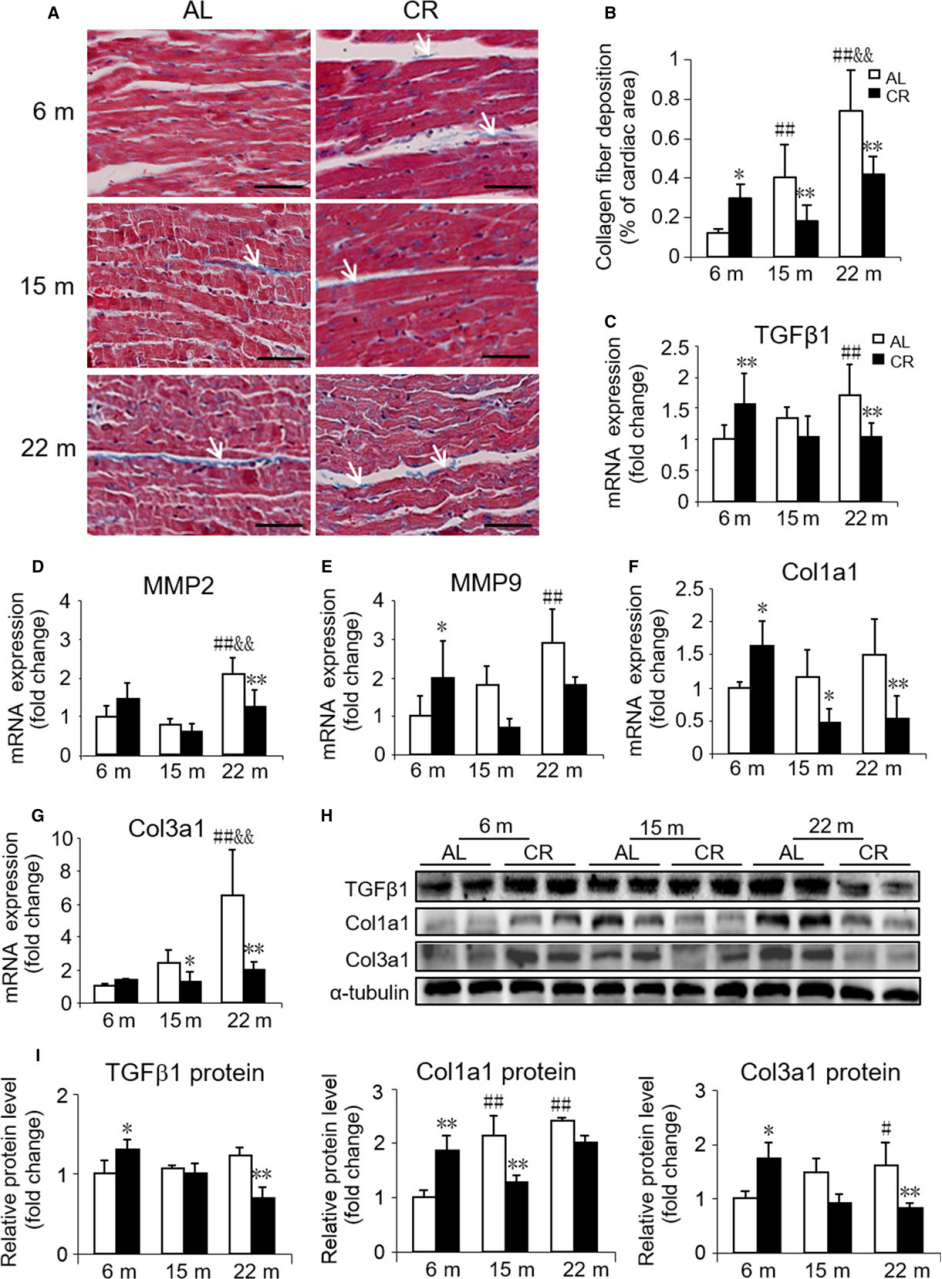
Effect of age and CR on cardiac fibrosis. (A) Representative images of Masson's trichrome staining of cardiac tissues from AL and CR mice at 6, 15, and 22 months of age (indicated by arrows). Scale bars, 50 μm. (B) Quantitation of cardiac fibrosis. (C‐G) mRNA quantification of the fibrosis marker genes. (H) Representative Western blots and (I) densitometric analysis of fibrosis markers TGFβ1, Col1a1, Col3a1, and α‐tubulin (loading control). Mean ± SEM,* n* = 10 sections for Masson's trichrome staining, *n* = 4 for Western blots. *P < 0.05 vs. age‐matched AL group, **P < 0.01 vs. age‐matched AL group. #*P* < 0.05 vs. 6 months AL group, ##*P* < 0.01 vs. 6 months AL group. &*P* < 0.05 vs. 15 months AL group, &&*P* < 0.01 vs. 15 months AL group.

As cardiac aging is usually accompanied with cardiac fibrosis, we further assessed the cardiac fibrosis in these mice. As expected, Masson's trichrome staining of heart tissue sections showed that the area of fibrosis was markedly increased with aging (Fig. 2A,B). In addition, the mRNA levels of collagen type III (*Col3a1*), matrix metalloproteinase 2 (*MMP2*), matrix metalloproteinase 9 (*MMP9*), as well as the major profibrogenic cytokine, transforming growth factor‐β1 (*TGF*β*1*), were elevated in old mice (Fig. 2C–G). These data were further substantiated by Western blotting, wherein the expression levels of Col1a1 and Col3a1 were elevated in old mice, while TGFβ1 did not exhibit any statistical difference (Fig. 2H,I). In middle‐aged mice, only higher protein level of Col1a1 was observed. In response to CR, the degree of cardiac fibrosis significantly alleviated in middle‐aged and old mice, especially in old mice, but aggravated in young mice (Fig. 2A,B). Simultaneously, the expression of genes and proteins, which positively correlates with cardiac fibrosis, was also downregulated by CR in middle‐aged and in old mice while upregulated in young mice (Fig. 2C–I).

### Effects of 3 months CR initiated in different ages on cardiac inflammation

**Figure 3:**
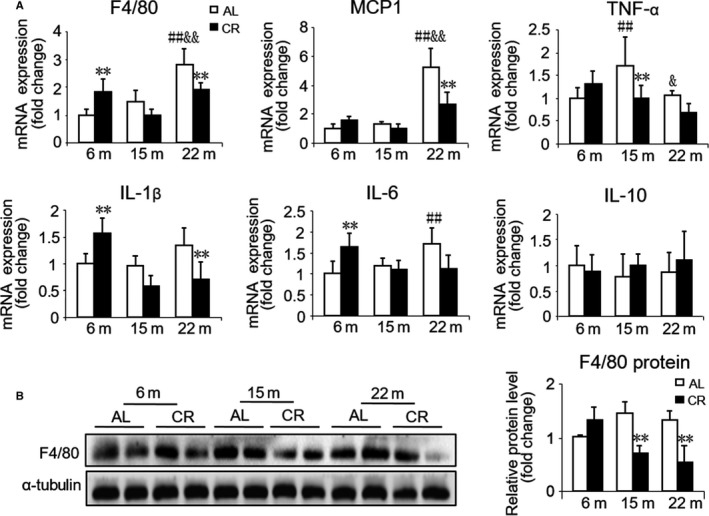
Effect of age and CR on cardiac inflammation. (A) mRNA quantification of the inflammation genes. (B) Representative Western blots (left) and densitometric analysis (right) of the specific macrophage marker F4/80 and α‐tubulin (loading control). Mean ± SEM,* n* = 4 for Western blots. *P < 0.05 vs. age‐matched AL group, **P < 0.01 vs. age‐matched AL group. #*P* < 0.05 vs. 6 months AL group, ##*P* < 0.01 vs. 6 months AL group. &*P* < 0.05 vs. 15 months AL group, &&*P* < 0.01 vs. 15 months AL group.

As inflammation appears to play a key role in aging and inflammatory cells have been observed to be associated with cardiac fibrosis, we next examined the expression of specific macrophage marker F4/80 and inflammation genes. The expression of pro‐inflammatory cytokines such as *F4*/*80*, monocyte chemoattractant protein‐1 (*MCP1*), *IL‐1*β, and *IL‐6* was higher in old mice while *TNF‐*α was higher in middle‐aged mice as compared to young mice. A significant change in the anti‐inflammatory cytokine *IL‐10* was not observed between mice at different ages. After CR, the gene expression of *F4*/*80, MCP1*, and *IL‐1*β significantly decreased in old mice, but the gene expression of *F4*/*80*,* IL‐1*β, and *IL‐6* increased in young mice. We also observed that the expression of *TNF‐*α decreased in middle‐aged mice after CR (Fig. 3A). Although there was no significant difference in protein expression of F4/80 between different‐aged mice, CR indeed downregulated the protein level of F4/80 both in middle‐aged and old mice (Fig. 3B).

### 3 months CR modulates many age‐related cardiac alterations

**Figure 4:**
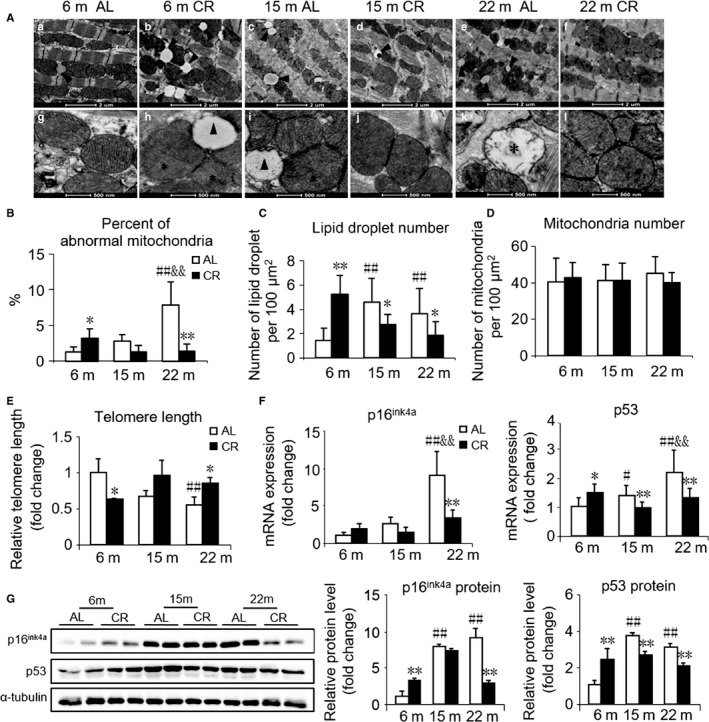
Effect of age and CR on cardiac mitochondrial structure, telomere length, and senescence markers. (A) Representative images of mitochondria in cardiac muscle from AL and CR mice at 6, 15, and 22 months of age using transmission electron microscopy. ‘*’ shows damaged mitochondria (mitochondria with reduced numbers of cristae and matrix density). ‘▲’ is labeling lipid droplets. a–f, scale bars, 2 μm. g–l, scale bars, 500 nm. (B) Quantitative morphometric analyses of cardiac mitochondrial ultrastructural abnormalities. (C) Quantification of lipid droplet number. (D) Quantification of mitochondrial number. (E) Telomere lengths of cardiac tissues from AL and CR mice at 6, 15, and 22 months of age by RT‐qPCR. (F) mRNA quantification of the senescence marker genes. (G) Representative Western blots (left) and densitometric analysis (right) of senescence markers p16^ink4a^, p53, and α‐tubulin (loading control). Mean ± SEM,* n* = 20 sections for the quantitative morphometric analysis, *n* = 10 for telomere length analysis, *n* = 4 for Western blots. *P < 0.05 vs. age‐matched AL group, **P < 0.01 vs. age‐matched AL group. #*P* < 0.05 vs. 6 months AL group, ##*P* < 0.01 vs. 6 months AL group. &*P* < 0.05 vs. 15 months AL group, &&*P* < 0.01 vs. 15 months AL group.

The high‐energy cardiac muscle is composed of a large number of mitochondria and is susceptible to oxidative damage. Using electron microscopy, we found that aging induced mitochondrial damage (mitochondria with reduced numbers of cristae and matrix density; Fig. 4A, g, i, k, asterisks), and the quantitative analysis showed a significant increase in the number of damaged mitochondria in old mice vs. other ages (Fig. 4B). Lipid droplets were rare in young mice; however, an increased number of droplets could be found in middle‐aged and old mice (Fig. 4A, a, c, e, triangles). In response to CR, not only the damaged mitochondria but also lipid droplets in the hearts of old mice reduced but increased in young mice, only reduced lipid droplets was observed in middle‐aged mice (Fig. 4A–C). We did not observe any significant changes in the number of cardiac mitochondria before and after CR in all groups (Fig. 4D).

Telomere shortening has been conventionally considered as a vital hallmark of aging. Using real‐time quantitative PCR (RT‐qPCR), we found that telomere length in the hearts shortened with aging. In young mice, telomere length was shortened by CR, while in old mice, it was lengthened by CR. To be noted, there was also an increase in telomere length in middle‐aged mice with CR, while there was no significant statistical difference (*P* *= *0.057, Fig. 4E). In line with the previous observation, mRNA as well as protein expression of p16^ink4a^ and p53, inducers of cellular senescence, was high in middle‐aged and old mice (Fig. 4F,G). After CR, the mRNA and protein expressions of the two cell cycle regulators significantly decreased in old mice but were dramatically increased in young mice. In middle‐aged mice, the decline of p53 was significant (Fig. 4F,G).

### Different effects of 3 months CR on cardiac aging involving autophagy

**Figure 5:**
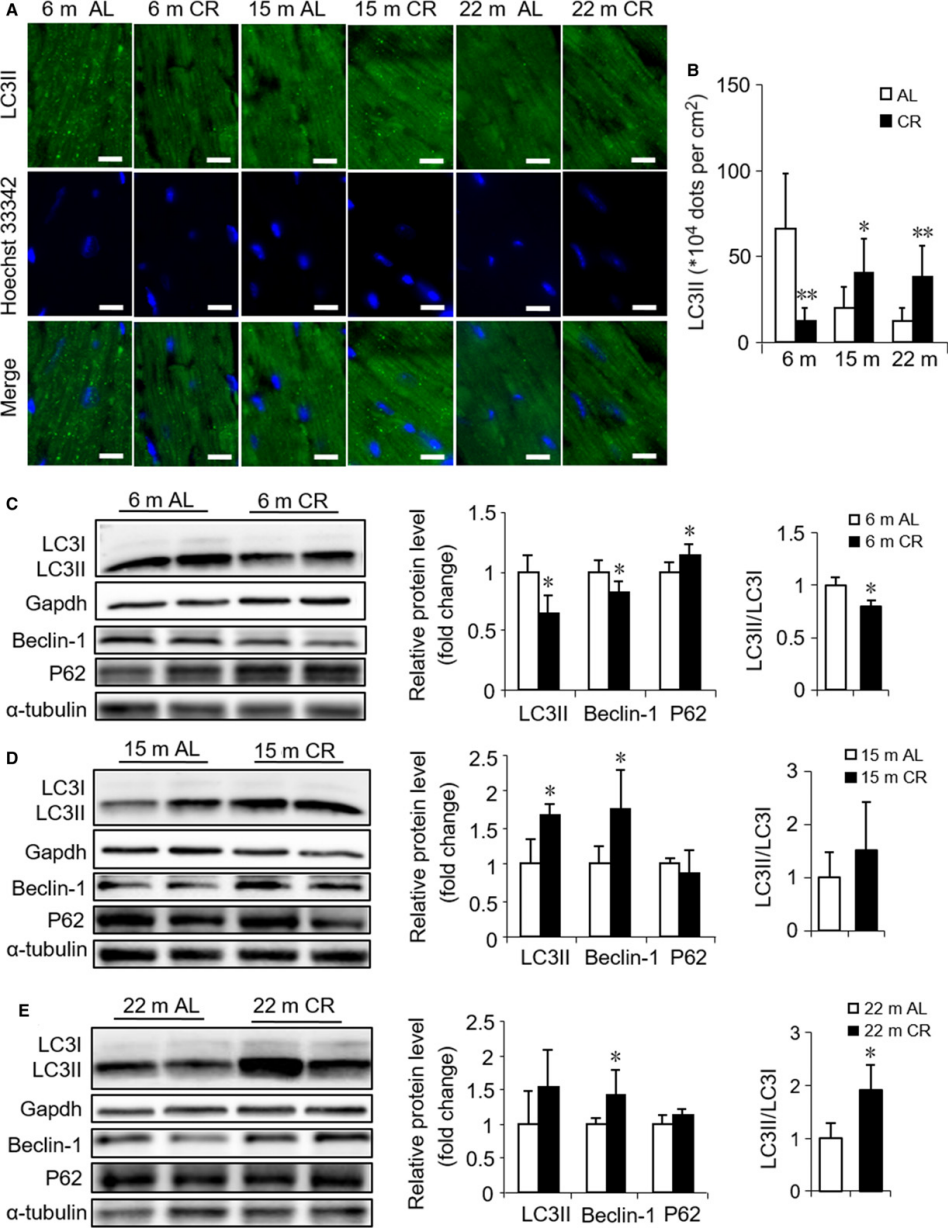
Different effects of CR initiated in different ages on cardiac autophagy. (A) Representative immunofluorescence images of LC3II protein in LV sections from AL and CR mice at 6, 15, and 22 months of age. Scale bars, 10 μm. (B) Quantitation of LC3II dots/field. (C, D, E) Representative Western blots (left) and densitometric analysis (right) of autophagy‐related markers LC3II, Beclin‐1, p62, LC3II/LC3I, and Gapdh (loading control for LC3II), α‐tubulin (loading control for Beclin‐1 and p62) in AL and CR mice at 6 months (C), 15 months (D), 22 months (E). Mean ± SEM,* n* = 4 for Western blots, *n* = 15 sections for immunofluorescence. *P < 0.05 vs. age‐matched AL group, **P < 0.01 vs. age‐matched AL group.

Autophagy is known as a protective cellular response to nutrient deprivation. Recent studies have revealed that the induction of autophagy is a downstream mediator of various life‐prolonging interventions (Finkel, 2015); thus, we further evaluated autophagy. Light chain 3 (LC3) conversion and sequestosomes (p62) protein degradation are two widely used indirect measurements of autophagic flux. Conversion of LC3‐I to LC3‐II is an indicator of autophagosome formation, and p62 is an autophagy‐specific substrate and degraded during autophagy. Therefore, a decrease in p62 abundance is considered indicative of enhanced autophagic flux (Morselli *et al*., 2010). Using immunofluorescence staining, we found that LC3II protein expression was downregulated by CR in young mice but was upregulated in middle‐aged and old mice, and these data were further substantiated by Western blotting although the difference of LC3II did not show statistical significance in old mice. Moreover, the LC3‐II/LC3‐I ratio decreased and the level of p62 expression increased after CR in young mice, which changed inversely in middle‐aged mice without statistical difference. The LC3‐II/LC3‐I ratio increased by CR in old mice, while the change in the p62 expression was not observed. Beclin‐1 is required for the induction phase of autophagy. We observed that the protein expression of Beclin‐1 was downregulated by CR in young mice, while upregulated in middle‐aged and old mice (Fig. 5). The present results suggested that CR accelerated autophagy in middle‐aged and old mice, but suppressed autophagy in young mice.

### Pathway analysis of 3 months CR initiated in different‐aged mice

**Figure 6:**
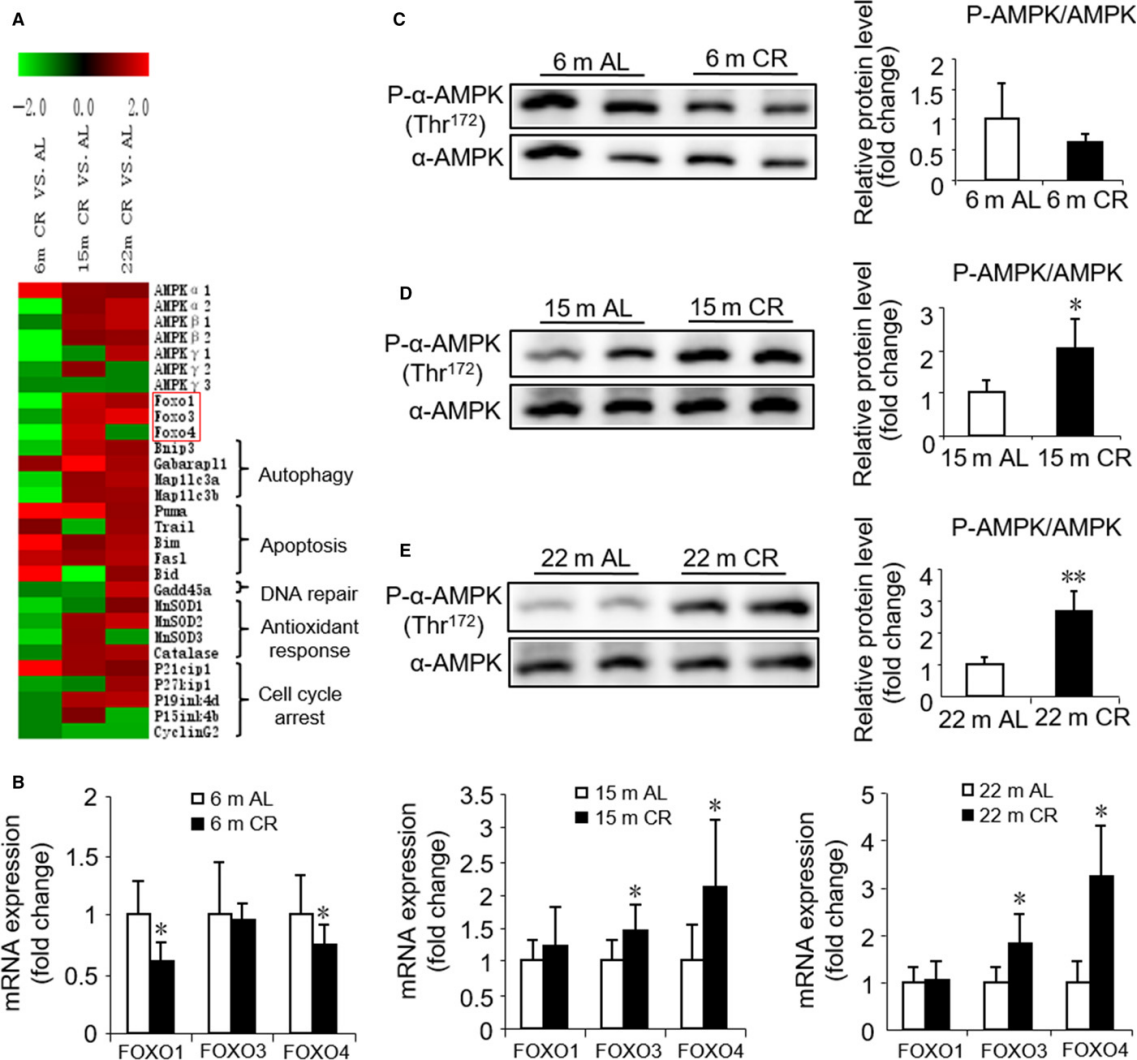
Transcriptional and post‐transcriptional modifications of the FOXO pathway in cardiac tissues upon CR initiation in different ages. (A) Heat map of expression changes of AMPK, FOXOs, and associated target genes in CR vs. AL. ‘Red’ indicates upregulation, and ‘green’ indicates downregulation. Red square represents genes tested and verified by RT‐qPCR. Significant cutoffs were set by fold change ≥ 2. (B) RT‐qPCR validation of differential mRNAs expression. (C, D, E) Representative Western blots (left) and densitometric analysis (right) of AMPK phosphorylation in AL and CR mice at 6 months (C), 15 months (D), 22 months (E). Mean ± SEM,* n* = 8 for RT‐qPCR,* n* = 4 for Western blots. *P < 0.05 vs. age‐matched AL group, **P < 0.01 vs. age‐matched AL group.

Several pieces of evidence suggested that lifespan is controlled by a large number of genes. Previous studies have shown that aging and CR involve a complex interplay between the genome and epigenome (Vermeij *et al*., 2016). To elucidate the possible molecular pathways underlying the observed different effects of CR on cardiac aging, we performed whole‐genome and miRNA microarray in myocardial tissue. The pathway analysis of differentially expressed genes (DEGs) demonstrated that 20, 15, and 4 signaling pathways were upregulated while 69, 24, and 18 signaling pathways were downregulated by CR in young, middle‐aged, and old mice, respectively (Table S1, Supporting information). Some of these altered pathways, such as p53, cell cycle, and TGFβ signaling pathways, were closely associated with cardiac aging. Notably, a heat map analysis of the genes involved in FOXO signaling pathway revealed significant differences between CR initiated in different‐aged mice, which was downregulated by CR initiated in young mice but upregulated in middle‐aged and old mice (Fig. 6A). As FOXO signaling pathway has been reported to be involved in the aging process and CR, we further assessed its gene expression profile changes, including FOXOs and their target genes. Remarkably, in the young mice, CR induced a significant decrease in *FOXO1* and *FOXO4* as compared to the age‐matched AL group (‐3.0‐, ‐2.05‐fold change, respectively). However, in middle‐aged mice, CR induced an increase in *FOXO1*,* FOXO3*, and *FOXO4* as compared to age‐matched AL group (1.54‐, 1.51‐, 1.63‐fold change, respectively), and in old mice, CR induced a 1.81‐fold increase in *FOXO3* (Table S2, Supporting information), which was further validated by RT‐qPCR (Fig. 6B). The activation of FOXO is known to modify several ‘longevity genes’ (i.e., autophagy, antioxidants, DNA repair, and cell death genes) (Lanvin *et al*., 2007). Importantly, we found that several transcripts downstream of FOXO inducing autophagy, such as BCL2/adenovirus E1B interacting protein 3 (*Bnip3*) and microtubule‐associated protein 1 light chain 3 beta (*Map1lC3B*, a.k.a. LC3), were downregulated (‐1.54‐,‐1.88‐fold change, respectively) by CR in young mice.

Recent studies suggested that the energy sensor AMPK could modulate FOXO transcription factor expression and subsequently regulate the expression of autophagy‐associated genes (Sanchez *et al*., 2012; Kubli & Gustafsson, 2014). We found that CR in young mice induces a significant decrease in mRNA level of *AMPK*α*2*,* AMPK*β*2*, and *AMPK*γ*1* as compared to the age‐matched AL group (‐2.12‐,‐2.41‐,‐4.17‐fold change, respectively) but not in middle‐aged and old mice (Table S2, Supporting information). We further assessed the AMPK activity and found that the level of phospho‐Thr172‐AMPKα was upregulated by CR in both middle‐aged and old mice (Fig. 6D,E); however, it was downregulated (without a significant statistical difference) in young mice (Fig. 6C). Thus, our data revealed that AMPK activation might be a major contributor to various regulations of FOXO signaling pathway by CR initiated in different ages, thereby leading to different regulation of autophagy.

In addition, we also found that 85, 161, and 58 miRNAs were upregulated while 82, 77, and 36 miRNAs were downregulated by CR in young, middle‐aged, and old mice, respectively (Table S3, Supporting information). Among these altered miRNAs, seven were upregulated and 62 downregulated in young mice, but changed inversely or remained unchanged in middle‐aged and old mice after CR; 16 miRNAs changed similarly (12 miRNAs were upregulated and four downregulated) both in middle‐aged and old mice after CR (Table S4, Supporting information, Fig. S2A, Supporting information). Nine miRNAs (miR‐2137, ‐1892, ‐146a/b‐5p, ‐19a‐3p, ‐34b/c‐5p,‐201‐5p, and ‐138‐2‐3p) with significant alterations in the expression level were further validated by RT‐qPCR (Fig. S2B, Supporting information). Furthermore, we found that AMPK, FOXO, and some aging‐related genes such as p21^cip1^ and RAD21 were predicted as target genes of some miRNAs both in miRDB and Target Scan database, and these miRNAs changed differently in the three age groups by CR (Table 2). The present study suggested that miRNAs were modulated differentially by CR initiated in different ages and, therefore, may regulate the expression of AMPK, FOXO, and aging‐related genes in opposite directions.

**Table 2:** Fold changes for some miRNAs expression modulated differentially by CR initiated in different ages and their predicted target genes both in miRDB and Target Scan database

miRNAs	CR group VS AL group (fold changes)			Target Genes
	Young (6 m)	Middle‐aged (15 m)	Old (22 m)	
mmu‐miR‐200c‐3p	1.73	N/A	−1.14	AMPKα2, Foxo3
mmu‐miR‐421‐3p	4.45	N/A	1.19	AMPKα2
mmu‐miR‐135b‐5p	19.3	N/A	1.28	Foxo1
mmu‐miR‐5127	1.69	N/A	1.13	Foxo4
mmu‐miR‐92a‐3p	−1.49	−1.61	−1.18	AMPKα2
mmu‐miR‐30d‐5p	−1.39	−1.54	−1.19	AMPKα2
mmu‐miR‐30d‐3p	N/A	N/A	−1.79	AMPKα2
mmu‐miR‐448‐3p	N/A	N/A	−4.17	AMPKβ2
let‐7b‐3p	1.18	N/A	−1.82	Foxo1
mmu‐miR‐328‐3p	−1.25	−1.64	−1.02	Foxo4
mmu‐miR‐674‐5p	1.06	1.54	−1.04	P21^cip1^
mmu‐miR‐302d‐3p	N/A	N/A	1.52	P21^cip1^
mmu‐miR‐302b‐3p	N/A	N/A	3.02	P21^cip1^
mmu‐miR‐883b‐5p	1.42	25.39	−1.2	P21^cip1^
mmu‐miR‐148a‐3p	−1.53	3.04	−1.16	Bim
mmu‐miR‐152‐3p	−1.66	3.13	−1.05	Bim
mmu‐miR‐466d‐5p	1.26	1.59	−1.07	Bim
mmu‐miR‐130a‐3p	−1.31	1.59	−1.28	Bim
mmu‐miR‐92b‐3p	1.29	11.79	1.75	Bim
mmu‐miR‐25‐3p	−1.44	1.55	−1.26	Bim
mmu‐miR‐24‐3p	−1.4	3.03	−1.44	Bim
mmu‐miR‐181d‐5p	−1.49	1.94	1.18	Bim
mmu‐miR‐214‐3p	−1.03	2.04	−1.03	Bim
mmu‐miR‐761	1.19	47.89	−1.04	Bim
mmu‐miR‐29b‐2‐5p	N/A	N/A	−2.38	RAD21
mmu‐miR‐345‐3p	5.45	N/A	−1.78	RAD21
mmu‐miR‐297a‐5p	2.07	N/A	−1.21	RAD21
mmu‐miR‐297c‐5p	3.18	N/A	1.05	RAD21
mmu‐miR‐297b‐5p	N/A	N/A	−20	RAD21
mmu‐miR‐205‐3p	2.29	N/A	1.44	RAD21
mmu‐miR‐574‐5p	1.43	−1.08	−1.66	RAD21
mmu‐miR‐292a‐3p	N/A	N/A	−3.33	RAD21

N/A, not available.

Differentially expressed (DE) miRNAs were identified using a threshold of fold change ≥ 1.5.

## Discussion

In the present study, we demonstrated age‐related cardiac changes including cardiomyocyte hypertrophy and cardiac fibrosis in 6‐, 15‐, and 22‐month‐old mice, which were in agreement with the previous remodeling of the aged heart (Boyle *et al*., 2011). CR is currently the only known intervention that significantly prolongs the maximal lifespan and retards the onset of age‐associated diseases. However, prior studies of CR were mostly concentrated in middle‐aged and elderly mice and the pathological states such as postmyocardial infarction models when CR initiated in 2‐ to 9‐month‐old mice (Peart *et al*., 2012; Dai *et al*., 2014; Noyan *et al*., 2015). The most significant finding of our study was that the age‐associated cardiomyocyte hypertrophy and cardiac fibrosis were attenuated by CR in middle‐aged and old mice, whereas both were exacerbated in young healthy mice.

Cardiomyocytes are the most energy‐demanding cells in the body, constantly contracting, 3 billion times or more in an average human lifespan, requiring large supplies of high‐energy phosphates. Therefore, mitochondrial dysfunctions are likely involved in the mechanisms contributing to the age‐related decline in cardiac function. Using transmission electron microscopy, we found that CR induced a loss of electron density and an increased lipid droplets infiltration in mitochondria in young mice, whereas a similar CR significantly improved age‐related mitochondrial damage and reduced lipid accumulation in middle‐aged and old mice. In healthy hearts, approximately 70–90% of the fatty acids taken up are immediately oxidized while only a small proportion is esterified to triacylglycerol (TAG) (Wisneski *et al*., 1987). Nevertheless, an accumulation of TAG has been associated with cardiac contractile dysfunction (Stanley *et al*., 2005). Furthermore, as two latest studies demonstrated, lipid accumulation may aggravate aging and impair the myocardial healing response by stimulating pro‐inflammatory mediators (Lopez *et al*., 2015; Halade *et al*., 2016). Although the mechanism underlying TAG accumulation remains unknown, a dysfunction in mitochondrial fatty acid oxidation may be potentially involved.

Another novel finding was that telomere shortening was observed during normal aging. Moreover, age‐related telomere shortening was retarded by CR in old mice but accelerated in young mice. Telomere shortening is known to be a critical hallmark of cellular senescence and organismal aging. Recent meta‐analyses have supported the existence of a strong correlation between short telomeres and mortality risk, particularly at younger ages (Liu *et al*., 2008). Additionally, the expressions of cell cycle regulators, p53 and p16^ink4a^, biomarkers of aging, were upregulated by CR in young mice but downregulated in middle‐aged and old mice. Thus, CR modulated the aging process involved in telomere length and expression of p53 and p16^ink4a^.

The mechanisms of aging and CR are complex. Our microarray data analysis demonstrated that several pathways are involved in the CR‐induced opposing effects on different‐aged mice. Pertinently, FOXOs and several of their target genes related with autophagy were altered in the opposite direction, that is, downregulated by CR initiated in young mice and upregulated when initiated in middle‐aged and old mice. In a recent study involving FOXO1^+/−^ and wild‐type mice with 30% CR, a partial loss of FOXO1 was observed in mice that did not respond to a CR‐specific reaction (Makino *et al*., 2016). A plethora of evidence suggested that FOXOs regulate various downstream target genes involved in the cell cycle, cell death, oxidative stress response, and autophagy (Accili & Arden, 2004). Autophagy is a protective cellular response to nutrient deprivation, whereby lysosomal enzymes mediate the recycling of proteins, cytoplasm, and cell organelles. Accumulating data showed that aging is associated with a decline in autophagy, and enhancing autophagy promotes longevity in both model organisms and rodents (Madeo *et al*., 2015). Transcriptional mechanisms are emerging that play crucial roles in the regulation of autophagy. Specifically, several transcription factors such as FOXO have been shown to be key transcriptional regulators of autophagy and lysosomal biogenesis (Fullgrabe *et al*., 2014). Consistent with the microarray data, the protein expression levels of Beclin‐1 and LC3II further suggested that autophagy was suppressed by CR in young mice while enhanced in middle‐aged and old mice. Notedly, LC3 conversion and p62 protein content, two indirect surrogates of flux, were measured in our study. Further studies should be performed to measure flux more directly to confirm our findings in future. A previous study showed that dysfunctional, senescent mitochondria accumulated in aging postmitotic cells when autophagy is suppressed (Terman *et al*., 2003). Hence, we observed damaged mitochondria accompanied by suppressed autophagy due to CR in young mice.

The energy sensor AMPK is activated by a decrease in energy levels, raising the probability that AMPK might mediate lifespan extension by CR (Greer *et al*., 2007a). In addition to phosphorylation and inhibition by protein kinase B (Akt), some studies suggested that FOXO1 and FOXO3 could be directly phosphorylated and activated by AMPK, resulting in enhanced cellular stress resistance (Greer *et al*., 2007b; Dixit *et al*., 2008). Other studies also reported that AMPK modulates FOXO transcription factor expression, which leads to the expression of autophagy‐associated genes (Sanchez *et al*., 2012; Kubli & Gustafsson, 2014). Herein, we confirmed that AMPK was phosphorylated and activated by CR in both middle‐aged and old mice, but the phosphorylation was suppressed by CR in young mice. Thus, we considered that AMPK might be a key junction as both an upstream energy sensor and a downstream autophagy activator. AMPK–FOXO–autophagy pathway may be a potential mechanism underlying the differential effects of CR in cardiac remodeling and function during aging. Accumulating evidence suggested that miRNAs are deeply involved in cardiac aging. In our study, some miRNAs were related to the effect of CR during aging by regulating pathways such as collagen synthesis, senescence‐associated inflammation, and cardiac fibrosis. For example, miR‐19a‐3p which was upregulated by CR in old mice was demonstrated as a novel regulator of collagen synthesis via CTGF and TSP‐1 (van Rooij, 2011). MiR‐146a‐5p and miR‐146b‐5p which could suppress the expression of senescence‐associated inflammatory mediators (IL‐6 and IL‐8) were upregulated by CR in old mice (Bhaumik *et al*., 2009). Additionally, aging‐related downregulation of miR‐146a induced expression of target gene NOX4, thus resulting in endothelial cell senescence (Touyz & Montezano, 2012). Moreover, miR‐101a‐5p and miR195a‐5p that were downregulated by CR in young mice have been reported to cause cardiac fibrosis via TGFβ pathway (Pan *et al*., 2012; Tijsen *et al*., 2014). In addition, some miRNAs were predicted to regulate the expression of AMPK, FOXO, and aging‐related genes. Due to the complex roles that miRNAs and pathways have exhibited in CR and aging, it may be too soon to draw any conclusions, and more efforts should be directed to investigate the relationships in the future.

There are a few studies when CR initiated in young healthy populations and the data are not uniform. Among these studies, the youngest model with beneficial effects of CR on cardiac function was established by 4‐month‐old mice for long‐term CR (20 weeks) (Han *et al*., 2012). There are also several studies demonstrating a neutral effect of CR on mitochondrial synthesis or myocardial ischemia. While these researches have potential limitations, CR was initiated too early (in 1‐month‐old rat) or lasted just 4 weeks (Hancock *et al*., 2011; Chen *et al*., 2013). To specifically study whether the effect of CR on heart function is suitable for three specific life phases of mice: mature adult (past development but not yet affected by senescence.), middle age (senescent changes can be detected in some biomarkers of aging), and old (a period when senescent changes can be detected in almost all biomarkers), we performed CR in different‐aged mice. Therefore, according to our research, if no senescence, there is detrimental rather than beneficial effects on young mice with CR by anti‐aging.

An issue requiring specific focus in this study is the degree and the time of CR. Despite the powerful effect of CR in increasing lifespan and preventing cardiac aging, a severe and prolonged CR is not only unpractical in humans but also associated with several adverse effects, such as slow wound healing, reduced reproductive function, increased risk of osteoporotic bone fractures, cardiac arrhythmias, and depression. Moreover, CR exceeding 50% typically causes starvation and increases mortality (Fontana *et al*., 2010). In 2006, Harper *et al*. reported that lower degree of CR would be beneficial especially in these growing animals (Harper *et al*., 2006). In our study, the daily caloric intake was restricted to 60% of the average *ad libitum* food intake for 3 months. Notedly, nutrition evaluation and graded levels of calorie restriction in young mice as well as CR on female mice merit further studies. Meanwhile, the heart rates in our young mice during echocardiograms were lower than optimal, considering that increased animal density per cage was found to be significantly positively correlated with behavioral acts of aggression and stress response (increased adrenocortical axis activity) (Whittaker *et al*., 2012). In future studies, it is worth confirming whether individual housing would affect heart rate compared to group housing in young mice.

As we know, the typical myocardial hypertrophy was characterized by increased organelles, enlarged mitochondria volume, elevated anabolism, and activated cell function. However, cardiomyocyte hypertrophy in our young CR mice was accompanied by mitochondrial matrix density decrease and/or fat droplet infiltration, which may contribute to the decline of heart weight. Even if so, some other mechanisms would be involved in the 20% decline of heart weight in our young CR mice, which need further research including the confirmation of the loss of cardiomyocytes by precise calculation of total number of cardiomyocytes per heart or per left ventricular.

Together, our findings highlight the significance of the initiation time of CR. Considering that 3 to 6 months of mice are equivalent to 20 to 30 years old of human, it was speculated that CR may have a detrimental effect on heart function of healthy men before 30 years old. From a translational therapeutic perspective, caloric restriction in the healthy, younger population who are robust healthy, vigorous vital, more fertile should be cautious. This result may propose an effective nutritional intervention for cardiac protection during aging.

## Materials and methods

### Animal model and diets

C57BL/6J male mice at different ages (Model Animal Research Center of Nanjing University) were maintained on a 12‐h light/dark cycle. Mice aged 3 months (young, *n = *22), 12 months (middle‐aged, *n = *22), or 19 months (old, *n* = 26) were caged individually and fed *ad libitum* with standard laboratory chow for 4 days. The average calorie intake was estimated from the daily food intake over these 4 days. Then, each age group was weight‐matched and randomly divided into two groups: fed *ad libitum* (AL) and calorie restricted (CR). For the next 12 weeks, the AL mice continued to be fed *ad libitum*. CR was progressive, being initiated at 10% restriction during the first week, changed to 25% during the second week, and to 40% for the remaining 10 weeks. During to the high content of minerals and vitamins in our diet formula, and even though 40% restriction is still meeting recommended intakes (Table S6, Supporting information), no micronutrient supplementation was added to mice during CR. Both groups had unlimited access to water. The food was provided at 10:00 a.m. daily. The food intake was monitored daily, and the animals were weighed weekly. At the end of the study, cardiac function was measured with echocardiography. The mice were sacrificed, and blood was withdrawn by cardiac puncture. The hearts were quickly removed, washed, weighed, and sliced into pieces for subsequent analyses. All tissues were stored at −80 °C until further analysis.

All protocols for animal usage were reviewed and approved by the Animal Care Committee of the Model Animal Research Center of Nanjing University and were in accordance with the Institutional Animal Care and Use Committee guidelines.

### Echocardiographic analysis

To examine the cardiac function, mice were lightly anesthetized with pentobarbital sodium (100 mg kg^−1^, i.p. injection). Two‐dimensional echocardiographic measurements were performed using the Vevo 770 system equipped with a 35 MHz transducer (Visual Sonics Inc., Toronto, ON, Canada). The parameters of cardiac function were measured digitally on the M‐mode tracings and averaged from at least five consecutive selected cardiac cycles (*n* = 6–10/group). All echocardiographic procedures were performed by a qualified investigator who was blinded to the treatment.

### Transmission electron microscopy

Detailed methods are provided in the supplement.

### Histopathological analysis

Detailed methods are provided in the supplement.

### RT‐qPCR of mRNA and miRNA

Detailed methods are provided in the supplement.

### Western blot analysis

Detailed methods are provided in the supplement.

### DNA isolation and measurement of telomere length by RT‐qPCR

Genomic DNA was extracted from the heart tissue using a Genomic DNA Isolation Kit (Invitrogen). The relative telomere length was measured using RT‐qPCR as described previously (Callicott & Womack, 2006). Detailed methods are provided in the supplement.

### Microarray analysis

Detailed methods are provided in the supplement.

### Statistics

Results were presented as mean ± SEM. Student's *t*‐test was used to compare two differences groups. One‐way ANOVA with Tukey post hoc analysis was used for comparisons between multiple groups using spss 20.0 software (SPSS Inc., Chicago, IL, USA). *P*‐value < 0.05 was considered significant.

## Funding

This research study was supported by the grants from the National Natural Science Foundation of China (91649122) to Guoxian Ding, the grants from the National Natural Science Foundation of China (81100236) to Shan Lv, and also by the National Natural Science Foundation of China (81501201), the Natural Science Foundation of Jiangsu Province of China (BK20151032) to Min Huang.

## Conflict of interest

None declared.

## Author contributions

GD, YS, and SL designed the experiments, conducted the study, wrote, and revised the manuscript. Protein assays, RT‐qPCR, and histopathological analysis were performed by YS and SL. Echocardiography and telomere length measurements were performed by MH and TT. Electron microscopy analysis was performed by JL. Microarray analysis was performed by YL, JY, HQ, and WD. GD is the guarantor of this work and, as such, has full access to all the data in the study and takes responsibility for the integrity of the data and the accuracy of the data analysis.

## Supporting information


**Fig. S1** Echocardiography of mice on AL and CR diets at 6, 15, and 22 months of age.
**Fig. S2** Differentially regulated miRNA levels in hearts by CR initiated in different ages.
**Table S1** Gene ontology pathway analysis of up‐regulated/down‐regulated DEGs by CR initiated in different ages.
**Table S2** Fold‐changes for mRNAs expression in cardiac tissues of mice by CR initiated in different ages (CR group vs. AL group).
**Table S3** Fold‐changes for miRNAs expression in cardiac tissues of mice by CR initiated in different ages (CR group vs. AL group).
**Table S4** Fold‐changes for miRNAs expression in cardiac tissues of mice differently regulated by CR initiated in different ages (CR group vs. AL group).
**Table S5** Sequences of primers used.
**Table S6** NRC micronutrient recommendations for mice versus the micronutrient contents provided by diet we used in 40% CR.
**Method S1** DNA isolation and measurement of telomere length by RT‐qPCR.
**Method S2** Microarray analysis.
**Method S3** Transmission electron microscopy.
**Method S4** Histopathological analysis.
**Method S5** RT‐qPCR of mRNA and miRNA.
**Method S6** Western blot analysis.
