# Supplementary material for: Opposing effects on cardiac function by calorie restriction in different‐aged mice
Source: Aging Cell. 2017 Aug 11;16(5):1155–67. doi: 10.1111/acel.12652 (PMC5595678; doi:10.1111/acel.12652)
Supplement: Supplementary file 1 — Fig. S1 Echocardiography of mice on AL and CR diets at 6, 15, and 22 months of age. Fig. S2 Differentially regulated miRNA levels in hearts by CR initiated in different ages. Table S1 Gene ontology pathway analysis of up‐regulated/down‐regulated DEGs by CR initiated in different ages. Table S2 Fold‐changes for mRNAs expression in cardiac tissues of mice by CR initiated in different ages (CR group vs. AL group). Table S3 Fold‐changes for miRNAs expression in cardiac tissues of mice by CR initiated in different ages (CR group vs. AL group). Table S4 Fold‐changes for miRNAs expression in cardiac tissues of mice differently regulated by CR initiated in different ages (CR group vs. AL group). Table S5 Sequences of primers used. Table S6 NRC micronutrient recommendations for mice versus the micronutrient contents provided by diet we used in 40% CR. Method S1 DNA isolation and measurement of telomere length by RT‐qPCR. Method S2 Microarray analysis. Method S3 Transmission electron microscopy. Method S4 Histopathological analysis. Method S5 RT‐qPCR of mRNA and miRNA. Method S6 Western blot analysis. [file ACEL-16-1155-s001.docx]

Supplementary Materials for

**Opposing effects on cardiac function by caloric restriction in different aged mice**

Yunlu Sheng^1*^, Shan Lv^1*^, Min Huang^1*^, Yifan Lv^1^, Jing Yu^1^, Juan Liu^1^, Tingting Tang^1^, Hanmei Qi^1^, Wenjuan Di^1^, and Guoxian Ding^1^

^*^These authors contributed equally to this research.

Corresponding authors: Guoxian Ding, MD, PhD, Department of Geratology, the First Hospital Affiliated to Nanjing Medical University, 300 Guangzhou Road, Nanjing, 210029, People’s Republic of China. Tel.: +86-25-83718836; fax: +86-25-83780170; e-mail: [dinggx@njmu.edu.cn](mailto:dinggx@njmu.edu.cn)

This pdf file includes:

**Fig.S1 to S2**

**Table S1 to S6**

**Method S1 to S6**

**
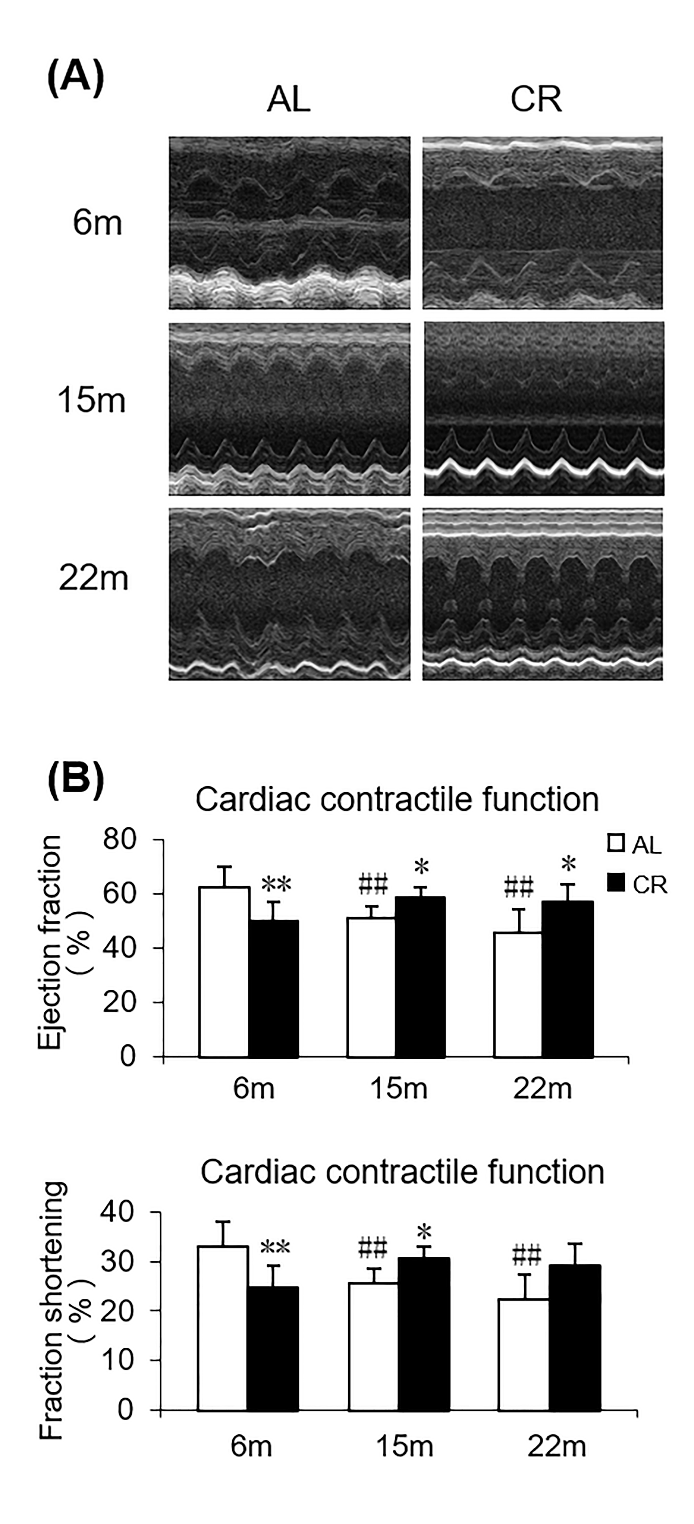
**

**Fig.S1** Echocardiography of mice on AL and CR diets at 6, 15, and 22 months of age. (A) Representative m-mode echocardiography. (B) Quantification of ejection fraction and fractional shortening. Mean ± SEM, n=6–10 mice per group. *P<0.05 vs. age-matched AL group, **P<0.01 vs. age-matched AL group. #P<0.05 vs. 6 months AL group, ##P<0.01 vs. 6 months AL group. &P<0.05 vs. 15 months AL group, &&P<0.01 vs. 15 months AL group.


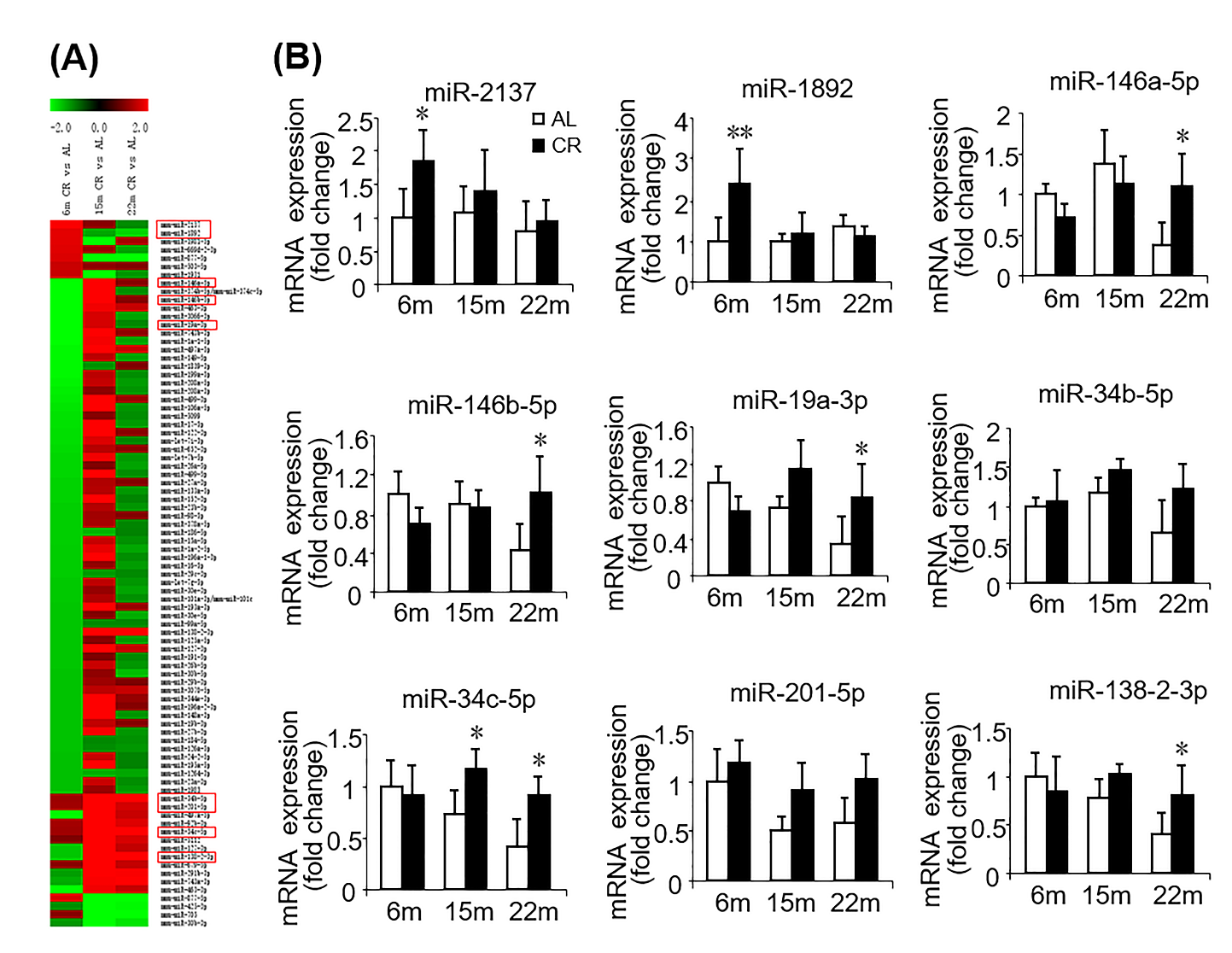


**Fig.S2** Differentially regulated miRNA levels in hearts by CR initiated in different ages. (A) Heat map of miRNAs differentially regulated by CR initiated in different ages. ‘‘Red’’ indicates up-regulation, and ‘‘green’’ indicates down-regulation. Red square represents miRNAs tested and verified by RT-qPCR. Significant cut-offs were set by fold change ≥1.5. (B) RT-qPCR validation of differentially regulated miRNAs. Rnu6 was used as an internal reference. Mean ± SEM, n=8 for RT-qPCR. *P<0.05 vs. age-matched AL group, **P<0.01 vs. age-matched AL group.

**Table S1** Gene ontology pathway analysis of up-regulated/down-regulated DEGs by CR initiated in different ages.

| **A. 6mCR vs. 6mAL Up DEGs** | | **B. 6mCR vs. 6mAL Down DEGs** | |
| --- | --- | --- | --- |
| **Ingenuity Canonical Pathways** | **Fisher-Pvalue** | **Ingenuity Canonical Pathways** | **Fisher-Pvalue** |
| Fatty acid metabolism | 0.00020966 | Proteoglycans in cancer | 3.83283E-06 |
| PPAR signaling pathway | 0.003385921 | Adrenergic signaling in cardiomyocytes | 4.01861E-05 |
| Fatty acid biosynthesis | 0.006897971 | Sphingolipid signaling pathway | 5.5133E-05 |
| T cell receptor signaling pathway | 0.008119469 | Thyroid hormone signaling pathway | 0.000364748 |
| Rap1 signaling pathway | 0.00894095 | Central carbon metabolism in cancer | 0.000580046 |
| Biosynthesis of unsaturated fatty acids | 0.01021146 | AMPK signaling pathway | 0.000669648 |
| Glutamatergic synapse | 0.01080198 | Osteoclast differentiation | 0.001043861 |
| Hedgehog signaling pathway | 0.01372332 | Choline metabolism in cancer | 0.001183395 |
| Cell cycle - Mus musculus | 0.01379143 | Regulation of actin cytoskeleton | 0.001233832 |
| Basal transcription factors | 0.01866596 | Prostate cancer | 0.003174162 |
| Steroid biosynthesis | 0.02708518 | Chagas disease (American trypanosomiasis) | 0.003554186 |
| Natural killer cell mediated cytotoxicity | 0.02735974 | Glycosphingolipid biosynthesis - ganglio series | 0.003607972 |
| Amino sugar and nucleotide sugar metabolism | 0.03567048 | Focal adhesion | 0.003616865 |
| Pathways in cancer | 0.03665649 | Bacterial invasion of epithelial cells | 0.004091941 |
| Peroxisome | 0.03997218 | Ubiquinone and other terpenoid-quinone biosynthesis | 0.004144786 |
| Transcriptional misregulation in cancer | 0.04232338 | PI3K-Akt signaling pathway | 0.004328231 |
| Purine metabolism | 0.04726218 | ErbB signaling pathway | 0.005697922 |
| Mismatch repair | 0.04863615 | Fc gamma R-mediated phagocytosis | 0.005697922 |
| Proximal tubule bicarbonate reclamation | 0.04863615 | FoxO signaling pathway | 0.005913999 |
| p53 signaling pathway | 0.04947164 | Butanoate metabolism | 0.006776783 |
|  |  | Apoptosis | 0.006954756 |
|  |  | MAPK signaling pathway | 0.007055702 |
|  |  | Inositol phosphate metabolism | 0.007338524 |
|  |  | TNF signaling pathway | 0.007831501 |
|  |  | Fc epsilon RI signaling pathway | 0.007980083 |
|  |  | Hepatitis B | 0.008525651 |
|  |  | Glioma | 0.009738077 |
|  |  | HTLV-I infection | 0.00985183 |
|  |  | Rap1 signaling pathway | 0.01002085 |
|  |  | Chronic myeloid leukemia | 0.01178852 |
|  |  | VEGF signaling pathway | 0.01189235 |
|  |  | cGMP-PKG signaling pathway | 0.01210246 |
|  |  | Adherens junction | 0.01333298 |
|  |  | N-Glycan biosynthesis | 0.01534389 |
|  |  | GnRH signaling pathway | 0.01567876 |
|  |  | Other types of O-glycan biosynthesis | 0.01623177 |
|  |  | Jak-STAT signaling pathway | 0.01796954 |
|  |  | Glycosaminoglycan biosynthesis - keratan sulfate | 0.01855111 |
|  |  | Insulin signaling pathway | 0.02058053 |
|  |  | Toxoplasmosis | 0.02073564 |
|  |  | Salmonella infection | 0.02113191 |
|  |  | Hepatitis C | 0.02290913 |
|  |  | Calcium signaling pathway | 0.02324839 |
|  |  | Amyotrophic lateral sclerosis (ALS) | 0.02350458 |
|  |  | Endometrial cancer | 0.02350458 |
|  |  | Axon guidance | 0.02354118 |
|  |  | Oxytocin signaling pathway | 0.02421245 |
|  |  | Synthesis and degradation of ketone bodies | 0.02537301 |
|  |  | Cardiac muscle contraction | 0.02613368 |
|  |  | Prolactin signaling pathway | 0.02888957 |
|  |  | Glucagon signaling pathway | 0.03061585 |
|  |  | Oxidative phosphorylation | 0.03110575 |
|  |  | Signaling pathways regulating pluripotency of stem cells | 0.03110575 |
|  |  | mTOR signaling pathway | 0.03140912 |
|  |  | p53 signaling pathway | 0.03191057 |
|  |  | Prion diseases | 0.0327464 |
|  |  | Viral carcinogenesis | 0.03315601 |
|  |  | Biotin metabolism | 0.0335329 |
|  |  | Valine, leucine and isoleucine degradation | 0.0344476 |
|  |  | Pathways in cancer | 0.03959069 |
|  |  | Dopaminergic synapse | 0.04009169 |
|  |  | Terpenoid backbone biosynthesis | 0.04120859 |
|  |  | Ubiquitin mediated proteolysis | 0.04134516 |
|  |  | Small cell lung cancer | 0.04242152 |
|  |  | Melanoma | 0.04360542 |
|  |  | Melanogenesis | 0.04405585 |
|  |  | Circadian rhythm | 0.04719846 |
|  |  | Dorso-ventral axis formation | 0.04936918 |
|  |  | HIF-1 signaling pathway | 0.04999091 |
|  |  |  |  |
| **C. 15mCR vs. 15mAL Up DEGs** | | **D. 15mCR vs. 15mAL Down DEGs** | |
| **Ingenuity Canonical Pathways** | **Fisher-Pvalue** | **Ingenuity Canonical Pathways** | **Fisher-Pvalue** |
| p53 signaling pathway | 0.000991826 | Platelet activation | 6.09145E-11 |
| GABAergic synapse | 0.01826625 | ECM-receptor interaction | 6.34954E-07 |
| Non-homologous end-joining | 0.01977513 | Hematopoietic cell lineage | 2.31704E-06 |
| Cell cycle | 0.02085593 | Focal adhesion | 7.79261E-06 |
| Base excision repair | 0.02112269 | Bacterial invasion of epithelial cells | 0.000124424 |
| Influenza A | 0.02624184 | Rap1 signaling pathway | 0.002365246 |
| FoxO signaling pathway | 0.02718727 | Regulation of actin cytoskeleton | 0.002737835 |
| Hepatitis C | 0.02805909 | Phagosome | 0.005263145 |
| Measles | 0.02894924 | Adherens junction | 0.005739881 |
| Tyrosine metabolism | 0.02999884 | Complement and coagulation cascades | 0.007288111 |
| Tight junction | 0.03173099 | Vascular smooth muscle contraction | 0.00806734 |
| Ether lipid metabolism | 0.03830501 | Malaria | 0.008899103 |
| TNF signaling pathway | 0.039014 | Hypertrophic cardiomyopathy (HCM) | 0.01210696 |
| Oocyte meiosis | 0.04723651 | Leukocyte transendothelial migration | 0.0138803 |
| Sphingolipid metabolism | 0.04765904 | Dilated cardiomyopathy | 0.01675947 |
|  |  | Arrhythmogenic right ventricular cardiomyopathy (ARVC) | 0.01906427 |
|  |  | Salmonella infection | 0.02473186 |
|  |  | Pantothenate and CoA biosynthesis | 0.02703199 |
|  |  | Viral myocarditis | 0.02795897 |
|  |  | Tryptophan metabolism | 0.02880133 |
|  |  | Rheumatoid arthritis | 0.03146071 |
|  |  | beta-Alanine metabolism | 0.03221398 |
|  |  | Pancreatic secretion | 0.0364139 |
|  |  | Proteoglycans in cancer | 0.04162013 |
|  |  |  |  |
| **E. 22mCR vs. 22mAL Up DEGs** | | **F. 22mCR vs. 22mAL Down DEGs** | |
| **Ingenuity Canonical Pathways** | **Fisher-Pvalue** | **Ingenuity Canonical Pathways** | **Fisher-Pvalue** |
| Primary bile acid biosynthesis | 0.01809711 | Cell cycle | 7.60691E-06 |
| Tryptophan metabolism | 0.02334189 | Oocyte meiosis | 0.000011974 |
| Retinol metabolism | 0.02904259 | Viral carcinogenesis | 3.14316E-05 |
| Drug metabolism - other enzymes | 0.03036373 | Basal transcription factors | 4.96893E-05 |
|  |  | RNA transport | 0.000795084 |
|  |  | TGF-beta signaling pathway | 0.000900785 |
|  |  | Ubiquitin mediated proteolysis | 0.001602157 |
|  |  | Epstein-Barr virus infection | 0.002865519 |
|  |  | p53 signaling pathway | 0.004778276 |
|  |  | Progesterone-mediated oocyte maturation | 0.006434731 |
|  |  | Biosynthesis of antibiotics | 0.007754926 |
|  |  | Glycolysis / Gluconeogenesis | 0.01576835 |
|  |  | Fanconi anemia pathway | 0.01991055 |
|  |  | Adherens junction | 0.02627148 |
|  |  | Herpes simplex infection | 0.02820771 |
|  |  | Protein processing in endoplasmic reticulum | 0.03618158 |
|  |  | RNA degradation | 0.04270905 |
|  |  | Vascular smooth muscle contraction | 0.04588301 |

Pathways analysis using the up- DEGs or down- DEG (fold-change ≥2) in CR vs AL.

**Table S2** Fold-changes for mRNAs expression in cardiac tissues of mice by CR initiated in different ages (CR group vs. AL group).

| **Gene name** | **CR group VS AL group（fold changes）** | | | |
| --- | --- | --- | --- | --- |
|  | **Young (6m)** | | **Middle-aged (15m)** | **Old (22m)** |
| AMPK family | |  |  |  |
| AMPKα1 | 1.91 | | 1.13 | 1.07 |
| AMPKα2 | -2.12 | | 1.09 | 1.49 |
| AMPKβ1 | -1.02 | | 1.2 | 1.49 |
| AMPKβ2 | -2.41 | | 1.06 | 1.17 |
| AMPKγ1 | -4.17 | | -1.1 | 1.42 |
| AMPKγ2 | -1.22 | | 1.12 | -1.04 |
| AMPKγ3 | -1.07 | | -1.08 | -1.04 |
| FOXO and its target genes | | |  |  |
| Foxo1 | -3.01 | | 1.54 | 1.33 |
| Foxo3 | -1.26 | | 1.51 | 1.81 |
| Foxo4 | -2.05 | | 1.63 | -1.08 |
| Bnip3 | -1.54 | | 1.48 | 1.2 |
| Gabarapl1 | 1.16 | | 2.21 | 1.31 |
| Map1lc3a | -1.65 | | 1.17 | 1.39 |
| Map1lc3b | -1.88 | | 1.2 | 1.23 |
| Puma | 7.45 | | 1.92 | 1.18 |
| Trail | 1.03 | | -1.39 | 1.22 |
| Bim | 2.17 | | 1.04 | 1.36 |
| Fasl | 1.53 | | 1.18 | 1.42 |
| Bid | 4.09 | | -2.52 | 1.12 |
| Gadd45a | -1.01 | | -1.14 | 1.54 |
| MnSOD1 | -1.64 | | -1.01 | 1.01 |
| MnSOD2 | -1.34 | | 1.22 | 1.55 |
| MnSOD3 | -1.65 | | 1.19 | -1.2 |
| Catalase | -1.06 | | 1.23 | 1.34 |
| P21^cip1^ | 3.25 | | 1.21 | 1.02 |
| P27^kip1^ | -1.25 | | -1.02 | 1.24 |
| P19^ink4d^ | -1.03 | | 1.36 | 1.44 |
| P15^ink4b^ | -1.03 | | 1 | -1.38 |
| CyclinG2 | -1.03 | | -1.36 | -1.36 |

AMPK, AMP-activated protein kinase; FOXO, Forkhead box subgroup ‘‘O’’; Bnip3, BCL2/adenovirus E1B interacting protein 3; Gabarapl1, gamma-aminobutyric acid (GABA) A receptor-associated protein-like 1; Map1lc3, microtubule-associated protein 1 light chain 3; Puma, BCL2 binding component 3; Trail, tumor necrosis factor (ligand) superfamily, member 10; Bim, BCL2-like 11; Fasl, Fas ligand; Bid, BH3 interacting domain death agonist; Gadd45a, growth arrest and DNA-damage-inducible 45 alpha; MnSOD, superoxide dismutase; P21^cip1^, cyclin-dependent kinase inhibitor 1A (P21); P27^kip1^, cyclin-dependent kinase inhibitor 1B(P27); P19^ink4d^, cyclin-dependent kinase inhibitor 2D (p19, inhibits CDK4); P15^ink4b^,cyclin-dependent kinase inhibitor 2B (p15, inhibits CDK4).

**Table S3** Fold-changes for miRNAs expression in cardiac tissues of mice by CR initiated in different ages (CR group vs. AL group).

| **A. 6mCR vs. 6mAL Up miRNAs** | | **B. 6mCR vs. 6mAL Down miRNAs** | |
| --- | --- | --- | --- |
| **name** | **fold change** | **name** | **fold change** |
| mmu-miR-592-3p | 58.86814932 | mmu-miR-146a-5p | -5.46005065 |
| mmu-miR-10a-5p | 42.14058777 | mmu-miR-374b-5p/mmu-miR-374c-5p | -3.5591698 |
| mmu-miR-5126 | 6.715150913 | mmu-miR-146b-5p | -3.54240597 |
| mmu-miR-712-5p | 5.437694752 | mmu-miR-463-3p | -3.00488924 |
| mmu-miR-10b-5p | 4.68228753 | mmu-miR-3066-3p | -2.41328785 |
| mmu-miR-421-3p | 4.454084438 | mmu-miR-144-3p | -2.29669875 |
| mmu-miR-466a-3p/mmu-miR-466e-3p | 4.161785147 | mmu-miR-142a-3p | -2.26846847 |
| mmu-miR-760-3p | 4.117553614 | mmu-miR-451a | -2.12040838 |
| mmu-miR-1946a | 4.079536429 | mmu-miR-767 | -2.10890522 |
| mmu-miR-1912-5p | 3.711737032 | mmu-miR-19a-3p | -2.09179647 |
| mmu-miR-743a-5p | 3.633135542 | mmu-miR-142a-5p | -2.06116479 |
| mmu-miR-3067-3p | 3.618943606 | mmu-miR-743b-3p | -2.02864429 |
| mmu-miR-3097-5p | 3.459619045 | mmu-miR-1a-1-5p | -1.99176462 |
| mghv-miR-M1-11-3p | 3.216838761 | mmu-let-7i-5p | -1.98632857 |
| mmu-miR-297c-5p | 3.176628276 | mmu-miR-497a-5p | -1.96880658 |
| mmu-miR-1934-5p | 3.113069769 | mmu-miR-149-5p | -1.96182087 |
| mmu-miR-215-3p | 2.939020868 | mmu-miR-1839-3p | -1.95457719 |
| mmu-miR-3552 | 2.646998752 | mmu-miR-199a-5p | -1.94901851 |
| mmu-miR-467h | 2.627909819 | mmu-miR-208a-5p | -1.92618811 |
| mmu-miR-5625-3p | 2.611585212 | mmu-miR-1949 | -1.87923554 |
| mcmv-miR-m108-2-5p.1 | 2.609213662 | mmu-miR-3064-3p | -1.87505389 |
| mmu-miR-504-3p | 2.425001527 | mmu-miR-208a-3p | -1.87297305 |
| mmu-miR-361-3p | 2.412629071 | mmu-miR-499-3p | -1.83522237 |
| mmu-miR-5135 | 2.329434965 | mmu-miR-106a-5p | -1.83437882 |
| mmu-miR-205-3p | 2.286752771 | mmu-miR-5099 | -1.79795463 |
| mmu-miR-325-5p | 2.261389636 | mmu-miR-17-5p | -1.77636684 |
| mmu-miR-302b-5p | 2.233405197 | mmu-miR-3071-5p | -1.75972435 |
| mmu-miR-490-5p | 2.229133339 | mmu-miR-378a-3p/mmu-miR-378b/mmu-miR-378c | -1.75529831 |
| mmu-miR-3064-5p | 2.214669762 | mmu-miR-122-3p | -1.74204091 |
| mmu-miR-5130 | 2.181281078 | mmu-let-7i-3p | -1.74083951 |
| mmu-miR-5119 | 2.171366164 | mmu-miR-652-3p | -1.73386302 |
| mmu-miR-5626-5p | 2.093979948 | mmu-let-7b-5p | -1.70526972 |
| mmu-miR-681 | 2.090945195 | mmu-miR-26a-5p | -1.69215468 |
| mmu-miR-297a-5p | 2.072022614 | mmu-miR-499-5p | -1.69136864 |
| mghv-miR-M1-5-5p | 2.035973899 | mmu-miR-126a-3p | -1.6894447 |
| mmu-miR-882 | 2.014624875 | mmu-miR-27a-3p | -1.67171027 |
| mmu-miR-29b-1-5p | 2.010524226 | mmu-miR-133a-5p | -1.6690855 |
| mmu-miR-141-5p | 1.983717236 | mmu-miR-301a-5p | -1.66582472 |
| mmu-miR-2137 | 1.966399446 | mmu-miR-152-3p | -1.65382498 |
| mmu-miR-365-2-5p | 1.957287809 | mmu-miR-378b | -1.6439795 |
| mmu-miR-3472 | 1.883027567 | mmu-miR-23b-3p | -1.63586919 |
| mghv-miR-M1-2-3p | 1.874158235 | mmu-miR-98-5p | -1.63251203 |
| mmu-miR-669h-3p | 1.867841861 | mmu-miR-378a-5p | -1.62980678 |
| mmu-miR-154-5p | 1.84237129 | mmu-miR-186-5p | -1.62833627 |
| mmu-miR-666-5p | 1.837564059 | mmu-miR-15a-5p | -1.62815899 |
| mmu-miR-711 | 1.819601162 | mmu-miR-1a-2-5p | -1.61558364 |
| mmu-miR-147-3p | 1.815376839 | mmu-miR-301b-5p | -1.6142027 |
| mmu-miR-1894-3p | 1.798505307 | mmu-miR-196a-1-3p | -1.61023181 |
| mmu-miR-1892 | 1.795248881 | mmu-miR-16-5p | -1.60603503 |
| mmu-miR-1981-5p | 1.794873092 | mmu-miR-29c-3p | -1.60529427 |
| mmu-miR-92a-2-5p | 1.792871144 | mmu-let-7g-5p | -1.59429838 |
| mmu-miR-3081-3p | 1.787538811 | mmu-miR-378a-3p | -1.5926832 |
| mmu-miR-669d-2-3p | 1.774135317 | mmu-miR-21a-5p | -1.5877676 |
| mmu-miR-466e-5p | 1.742902606 | mmu-miR-30e-3p | -1.58373732 |
| mmu-miR-877-5p | 1.732094656 | mmu-miR-101a-3p/mmu-miR-101c | -1.58175385 |
| mmu-miR-1899 | 1.725395335 | mmu-miR-133b-3p | -1.57309637 |
| mmu-miR-5108 | 1.715647339 | mmu-miR-193a-3p | -1.57178527 |
| mmu-miR-5127 | 1.694724811 | mmu-miR-30e-5p | -1.57152319 |
| mmu-miR-5132-5p | 1.670772202 | mmu-miR-99a-5p | -1.56856806 |
| mmu-miR-344c-5p | 1.668886274 | mmu-let-7a-5p | -1.56526691 |
| mmu-miR-124-3p | 1.642641069 | mmu-miR-133a-3p | -1.55911977 |
| mmu-miR-125a-3p | 1.639350522 | mmu-miR-138-2-3p | -1.55715217 |
| mmu-miR-1187 | 1.633164294 | mmu-miR-125a-5p | -1.55532758 |
| mmu-miR-468-3p | 1.627934449 | mmu-miR-127-3p | -1.55432099 |
| mmu-miR-105 | 1.625350111 | mmu-miR-191-5p | -1.55278358 |
| mmu-miR-1967 | 1.62373766 | mmu-miR-26b-5p | -1.55170956 |
| mmu-miR-471-5p | 1.622559331 | mmu-miR-30b-5p | -1.54749468 |
| mmu-miR-1964-5p | 1.620558395 | mmu-miR-29b-3p | -1.54570638 |
| mmu-miR-1946b | 1.617880671 | mmu-miR-3070-5p | -1.54358302 |
| mmu-miR-466m-3p | 1.605136892 | mmu-miR-344e-3p | -1.54269265 |
| mmu-miR-1968-3p | 1.597326833 | mmu-miR-196a-2-3p | -1.54056593 |
| mmu-miR-503-5p | 1.594572061 | mmu-miR-148a-3p | -1.53917166 |
| mmu-miR-206-3p | 1.584356413 | mmu-miR-19b-3p | -1.53760786 |
| mmu-miR-3104-3p | 1.577035588 | mmu-miR-27b-3p | -1.53744215 |
| mmu-miR-3470a | 1.576177716 | mmu-miR-184-5p | -1.53596699 |
| mmu-miR-1931 | 1.567994376 | mmu-miR-22-3p | -1.53499915 |
| mghv-miR-M1-6-3p | 1.565322976 | mmu-miR-126a-5p | -1.53287535 |
| mmu-miR-721 | 1.560866112 | mmu-miR-24-2-5p | -1.51731335 |
| mmu-miR-1962 | 1.551651638 | mmu-miR-195a-5p | -1.51552002 |
| mmu-miR-3099-3p | 1.544082605 | mmu-miR-1264-3p | -1.5125736 |
| mmu-miR-466n-5p | 1.515663907 | mmu-miR-23a-3p | -1.50661422 |
| mmu-miR-1927 | 1.508892877 | mmu-miR-1983 | -1.50541255 |
| mmu-miR-465c-5p | 1.507318734 |  |  |
| mmu-miR-219a-2-3p | 1.507148531 |  |  |
| mmu-miR-669a-5p/mmu-miR-669p-5p | 1.50548054 |  |  |
|  |  |  |  |
| **C. 15mCR vs. 15mAL Up miRNAs** | | **D. 15mCR vs. 15mAL Down miRNAs** | |
| **name** | **fold change** | **name** | **fold change** |
| mmu-miR-490-5p | 196.6908367 | mmu-miR-130b-3p | -4.94847133 |
| mmu-miR-1843b-5p | 126.8808765 | mmu-miR-3092-3p | -4.4895959 |
| mmu-miR-1899 | 109.5557769 | mmu-miR-877-5p | -4.39768227 |
| mcmv-miR-M23-1-5p | 71.33864542 | mmu-miR-5128 | -4.3608795 |
| mmu-miR-28b | 70.93099602 | mmu-miR-425-3p | -4.25832493 |
| mmu-miR-34b-5p | 65.90332005 | mmu-miR-125b-1-3p | -4.20529431 |
| mmu-miR-1843a-5p | 58.09003984 | mmu-miR-714 | -4.02249107 |
| mmu-miR-335-5p | 50.4858106 | mmu-miR-705 | -3.99816788 |
| mmu-miR-761 | 47.89880478 | mmu-miR-142a-3p | -3.77693212 |
| mmu-miR-743b-3p | 36.80168216 | mmu-miR-346-3p | -3.62467262 |
| mmu-miR-148b-3p | 30.80532416 | mmu-miR-3078-3p | -3.48318999 |
| mmu-miR-296-3p | 30.57370518 | mmu-miR-678 | -3.38471067 |
| mmu-miR-883b-5p | 25.39316069 | mmu-miR-290a-5p | -3.29697076 |
| mmu-miR-29c-5p | 25.07043825 | mmu-miR-194-2-3p | -3.16727863 |
| mmu-miR-137-5p | 23.6339594 | mmu-miR-744-5p | -3.14606281 |
| mmu-miR-7b-3p | 22.42071713 | mmu-miR-1981-5p | -3.13362254 |
| mmu-miR-201-5p | 22.31880478 | mmu-miR-1971 | -3.05899594 |
| mmu-miR-155-5p | 20.14267635 | mmu-miR-3474 | -3.04401008 |
| mmu-miR-34c-3p | 19.4832435 | mmu-miR-291b-5p | -3.01321019 |
| mmu-miR-374c-3p | 18.62957769 | mmu-miR-1931 | -2.99644036 |
| mmu-miR-497a-5p | 17.92119071 | mmu-miR-1198-5p | -2.99559827 |
| mmu-miR-499-3p | 13.0356544 | mmu-miR-103-3p | -2.94284593 |
| mmu-miR-1193-3p | 12.50124834 | mmu-miR-1954 | -2.93859542 |
| mmu-miR-129-1-3p | 12.1240555 | mmu-miR-5120 | -2.86811667 |
| mmu-miR-147-3p | 11.88977424 | mmu-miR-107-3p | -2.84678308 |
| mmu-miR-92b-3p | 11.79271485 | mmu-miR-344f-5p | -2.80929022 |
| mmu-miR-465a-5p | 11.21035857 | mmu-miR-767 | -2.79046771 |
| mmu-miR-190a-5p | 11.04397105 | mmu-miR-291a-5p | -2.75360367 |
| mmu-miR-3103-5p | 9.279387712 | mmu-miR-673-3p | -2.65036397 |
| mmu-miR-466m-5p/mmu-miR-669m-5p | 8.611593625 | mmu-miR-695 | -2.60698737 |
| mmu-miR-122-3p | 8.08994948 | mmu-miR-142a-5p | -2.60053601 |
| mmu-miR-301a-3p | 7.287431098 | mmu-miR-30b-3p | -2.53360957 |
| mmu-miR-5627-5p | 6.820288078 | mmu-miR-300-3p | -2.50560517 |
| mmu-miR-146a-5p | 6.512199203 | mmu-miR-675-5p | -2.44970478 |
| mmu-miR-466a-5p | 6.336289624 | mmu-miR-27a-5p | -2.35629983 |
| mmu-miR-677-5p | 5.672226882 | mmu-miR-344b-5p | -2.30184531 |
| mmu-miR-466n-3p | 5.470467555 | mmu-miR-22-3p | -2.28430616 |
| mmu-miR-212-3p | 5.375682005 | mmu-miR-3960 | -2.23803475 |
| mmu-miR-541-3p | 5.205594887 | mmu-miR-1843b-3p | -2.22219997 |
| mmu-let-7c-1-3p | 5.084293935 | mmu-miR-470-5p | -2.19652682 |
| mmu-miR-193a-3p | 5.035346785 | mmu-miR-185-3p | -2.17877506 |
| mghv-miR-M1-10-3p | 4.945065194 | mmu-miR-207 | -2.12074464 |
| mmu-miR-34c-5p | 4.799145237 | mmu-miR-762 | -2.1063852 |
| mmu-miR-466j | 4.793065239 | mmu-miR-339-5p | -2.08764293 |
| mmu-miR-10a-5p | 4.667698268 | mmu-miR-223-3p | -2.05446149 |
| mmu-miR-329-3p | 4.655829793 | mmu-miR-21a-5p | -1.94891929 |
| mmu-miR-376c-3p | 4.605654306 | mmu-miR-5100 | -1.9394555 |
| mmu-miR-28a-5p/mmu-miR-28c | 4.429267545 | mmu-miR-491-3p | -1.91022692 |
| mmu-miR-3470a | 4.225634049 | mmu-miR-1956 | -1.88535462 |
| mmu-miR-146b-5p | 4.220032546 | mmu-let-7e-5p | -1.86404115 |
| mmu-miR-5112 | 4.166416686 | mmu-miR-706 | -1.85088755 |
| mmu-miR-196a-1-3p | 4.122086391 | mmu-miR-1947-3p | -1.79731226 |
| mmu-miR-15b-5p | 4.038324604 | mmu-miR-451a | -1.7873095 |
| mmu-miR-199a-3p/mmu-miR-199b-3p | 4.028437794 | mmu-miR-133b-3p | -1.76307355 |
| mmu-miR-127-3p | 3.919705792 | mmu-miR-3572-3p | -1.752329 |
| mmu-miR-20b-5p | 3.850022134 | mmu-miR-882 | -1.73457086 |
| mmu-miR-125b-2-3p | 3.817910448 | mmu-miR-5113 | -1.69139522 |
| mmu-miR-3084-3p | 3.747654355 | mmu-miR-344-5p | -1.67839458 |
| mmu-miR-466d-3p | 3.729368987 | mmu-miR-5116 | -1.67160128 |
| mmu-miR-151-5p | 3.70930982 | mmu-miR-335-3p | -1.66776211 |
| mmu-miR-5622-5p | 3.708769048 | mmu-miR-25-5p | -1.66560012 |
| mmu-miR-105 | 3.690879724 | mghv-miR-M1-8-5p | -1.66455197 |
| mmu-miR-139-5p | 3.633037579 | mmu-miR-301a-5p | -1.6621101 |
| mmu-miR-92a-2-5p | 3.513294192 | mmu-miR-320-5p | -1.66176953 |
| mmu-miR-3470b | 3.350754558 | mmu-miR-294-5p | -1.65028902 |
| mmu-miR-494-5p | 3.288989497 | mmu-let-7c-5p | -1.64292487 |
| mmu-miR-467b-3p | 3.276733425 | mmu-miR-3071-5p | -1.63254498 |
| mmu-miR-24-1-5p | 3.22938818 | mmu-miR-328-3p | -1.62867801 |
| mmu-let-7a-2-3p | 3.200405396 | mmu-miR-883a-5p | -1.60834063 |
| mmu-let-7f-1-3p | 3.195770006 | mmu-miR-92a-3p | -1.60629774 |
| mmu-miR-152-3p | 3.130351229 | mghv-miR-M1-5-5p | -1.59984023 |
| mmu-miR-17-5p | 3.100869692 | mmu-miR-29a-3p | -1.59085844 |
| mmu-miR-196a-2-3p | 3.08350189 | mmu-miR-378a-3p/mmu-miR-378b/mmu-miR-378c | -1.58603146 |
| mmu-miR-23a-5p | 3.057370518 | mmu-miR-3473b | -1.58536422 |
| mmu-miR-148a-3p | 3.040321983 | mmu-miR-222-3p | -1.55590669 |
| mmu-miR-466c-5p | 3.03849786 | mmu-miR-30d-5p | -1.52851831 |
| mmu-miR-138-2-3p | 3.036366324 | mmu-miR-1897-5p | -1.5256234 |
| mmu-miR-24-3p | 3.027992682 |  |  |
| mmu-miR-679-5p | 3.014907039 |  |  |
| mmu-miR-29a-5p | 3.010555657 |  |  |
| mmu-miR-468-3p | 2.896952929 |  |  |
| mmu-miR-3090-5p | 2.866284861 |  |  |
| mmu-miR-30a-3p | 2.865284412 |  |  |
| mmu-miR-363-5p | 2.853545817 |  |  |
| mmu-miR-499-5p | 2.839277462 |  |  |
| mmu-miR-344e-3p | 2.780240442 |  |  |
| mmu-miR-467c-3p | 2.756859741 |  |  |
| mmu-miR-467e-5p | 2.675880435 |  |  |
| mmu-miR-195a-5p | 2.471843947 |  |  |
| mmu-miR-1948-5p | 2.426984844 |  |  |
| mmu-miR-5129-5p | 2.422506695 |  |  |
| mmu-miR-291b-3p | 2.408236745 |  |  |
| mmu-miR-5616-5p | 2.404086219 |  |  |
| mmu-miR-3099-5p | 2.39932012 |  |  |
| mmu-miR-467d-3p | 2.392454572 |  |  |
| mmu-miR-135a-5p | 2.392382593 |  |  |
| mmu-miR-22-5p | 2.372298942 |  |  |
| mmu-miR-183-3p | 2.366182794 |  |  |
| mmu-miR-1936 | 2.354744995 |  |  |
| mmu-miR-28a-5p | 2.347758595 |  |  |
| mmu-miR-206-3p | 2.342812657 |  |  |
| mmu-miR-331-3p | 2.226356257 |  |  |
| mmu-miR-374b-5p/mmu-miR-374c-5p | 2.178636475 |  |  |
| mmu-miR-106a-5p | 2.152837454 |  |  |
| mmu-miR-30c-2-3p | 2.150102031 |  |  |
| mmu-miR-125a-3p | 2.115161994 |  |  |
| mmu-miR-214-3p | 2.041436757 |  |  |
| mmu-miR-361-5p | 2.029124423 |  |  |
| mghv-miR-M1-2-5p | 2.027288695 |  |  |
| mmu-miR-342-5p | 1.989132626 |  |  |
| mmu-miR-345-5p | 1.974244086 |  |  |
| mmu-miR-743a-3p | 1.972719612 |  |  |
| mmu-miR-298-5p | 1.959177085 |  |  |
| mmu-miR-27b-3p | 1.942636278 |  |  |
| mmu-miR-181d-5p | 1.941305996 |  |  |
| mmu-miR-551b-5p | 1.930637698 |  |  |
| mmu-miR-615-3p | 1.930534771 |  |  |
| mmu-miR-1a-1-5p | 1.926797123 |  |  |
| mmu-miR-463-3p | 1.912865173 |  |  |
| mmu-let-7b-5p | 1.906780285 |  |  |
| mmu-miR-106b-3p | 1.899590753 |  |  |
| mmu-miR-1187 | 1.883931781 |  |  |
| mmu-miR-3076-3p | 1.875620921 |  |  |
| mmu-miR-466i-3p | 1.869301404 |  |  |
| mmu-miR-199b-5p | 1.851216855 |  |  |
| mghv-miR-M1-6-3p | 1.830262623 |  |  |
| mmu-miR-1a-2-5p | 1.82451605 |  |  |
| mmu-miR-374b-5p | 1.81752775 |  |  |
| mmu-miR-711 | 1.76361308 |  |  |
| mmu-miR-544-5p | 1.760568567 |  |  |
| mmu-miR-700-5p | 1.753840452 |  |  |
| mmu-miR-99b-5p | 1.740111814 |  |  |
| mmu-let-7i-3p | 1.738908045 |  |  |
| mmu-miR-709 | 1.732304205 |  |  |
| mmu-miR-3066-3p | 1.697742675 |  |  |
| mmu-miR-19a-3p | 1.683690217 |  |  |
| mmu-miR-686 | 1.659902878 |  |  |
| mmu-miR-423-3p | 1.650009486 |  |  |
| mmu-miR-5624-3p | 1.624896219 |  |  |
| mmu-miR-3962 | 1.614604686 |  |  |
| mmu-miR-669o-5p | 1.612448835 |  |  |
| mmu-miR-3964 | 1.598864423 |  |  |
| mmu-miR-130a-3p | 1.596583441 |  |  |
| mmu-miR-24-2-5p | 1.590351968 |  |  |
| mmu-miR-23a-3p | 1.588690353 |  |  |
| mmu-miR-466d-5p | 1.586709889 |  |  |
| mmu-miR-466b-5p/mmu-miR-466o-5p | 1.586216425 |  |  |
| mmu-miR-26b-5p | 1.57971626 |  |  |
| mmu-miR-15a-5p | 1.576232652 |  |  |
| mmu-miR-693-5p | 1.56652157 |  |  |
| mmu-miR-487b-5p | 1.563962611 |  |  |
| mghv-miR-M1-12-3p | 1.562982685 |  |  |
| mmu-miR-194-1-3p | 1.556479536 |  |  |
| mmu-miR-10b-5p | 1.552822395 |  |  |
| mmu-miR-208a-5p | 1.550778146 |  |  |
| mmu-miR-25-3p | 1.548641986 |  |  |
| mmu-miR-674-5p | 1.539526998 |  |  |
| mmu-miR-1960 | 1.535419555 |  |  |
| mmu-miR-19b-3p | 1.526373273 |  |  |
| mmu-miR-201-3p | 1.52146767 |  |  |
| mmu-let-7g-5p | 1.504280528 |  |  |
|  |  |  |  |
| **E. 22mCR vs. 22mAL Up miRNAs** | | **F. 22mCR vs. 22mAL Down miRNAs** | |
| **name** | **fold change** | **name** | **fold change** |
| mmu-miR-471-3p | 42.91615854 | mmu-miR-582-5p | -81.0417362 |
| mmu-miR-202-5p | 17.4027977 | mmu-miR-877-5p | -2.89309277 |
| mmu-miR-449a-5p | 12.15138368 | mmu-miR-361-3p | -2.51590489 |
| mmu-miR-880-5p | 7.597073171 | mmu-miR-705 | -2.24009713 |
| mmu-miR-487b-3p | 6.848323171 | mmu-miR-880-3p | -2.1903172 |
| mmu-miR-34b-5p | 5.664662376 | mghv-miR-M1-2-3p | -2.05676127 |
| mmu-miR-760-5p | 4.270997246 | mmu-miR-218-5p | -2.02775459 |
| mmu-miR-380-5p | 3.56547619 | mmu-miR-425-3p | -1.95768547 |
| mmu-miR-493-5p | 3.148654331 | mmu-miR-192-5p | -1.9513735 |
| mmu-miR-302b-3p | 3.021958479 | mmu-miR-30b-3p | -1.82830644 |
| mmu-miR-34c-5p | 2.867050257 | mmu-miR-296-3p | -1.77963272 |
| mmu-miR-292a-5p | 2.591257416 | mmu-miR-30d-3p | -1.77415693 |
| mmu-miR-1899 | 2.438097184 | mmu-miR-345-3p | -1.77216573 |
| mmu-miR-759 | 2.35886687 | mmu-miR-133b-3p | -1.76694981 |
| mmu-miR-5709-5p | 2.28277439 | mmu-let-7a-5p | -1.7484231 |
| mghv-miR-M1-7-3p | 2.229062052 | mmu-miR-144-5p | -1.73796908 |
| mmu-miR-344-5p | 2.214776855 | mmu-miR-486a-3p | -1.7321386 |
| mmu-miR-465c-5p | 2.135498623 | mmu-miR-133a-3p | -1.72629319 |
| mmu-miR-743a-3p | 2.072251863 | mmu-miR-3552 | -1.70358004 |
| mmu-miR-3109-5p | 2.071200178 | mmu-miR-1892 | -1.69889988 |
| mmu-miR-340-3p | 2.061368178 | mmu-miR-451a | -1.68135472 |
| mmu-miR-20a-3p | 2.061019164 | mmu-miR-574-5p | -1.65757553 |
| mmu-miR-138-2-3p | 2.058804073 | mmu-miR-5108 | -1.6427379 |
| mmu-miR-666-5p | 2.054496951 | mmu-miR-5626-5p | -1.62752736 |
| mmu-miR-883b-3p | 2.029862695 | mmu-miR-690 | -1.62650537 |
| mmu-miR-3066-5p | 1.982994737 | mmu-miR-3473a | -1.60138069 |
| mmu-miR-291b-3p | 1.978767681 | mmu-miR-128-3p | -1.57052964 |
| mmu-miR-138-1-3p | 1.90922949 | mmu-miR-677-5p | -1.56633148 |
| mmu-miR-221-5p | 1.887319793 | mmu-miR-144-3p | -1.55360248 |
| mmu-miR-452-3p | 1.881559497 | mmu-miR-804 | -1.55348488 |
| mmu-miR-742-3p | 1.874846659 | mmu-miR-2861 | -1.54832767 |
| mmu-miR-183-5p | 1.797 | mmu-miR-126a-3p | -1.53223019 |
| mmu-miR-196b-3p | 1.796281487 | mmu-miR-466b-5p/mmu-miR-466o-5p | -1.51881763 |
| mmu-miR-741-3p | 1.794732969 | mmu-miR-669c-3p | -1.51204676 |
| mmu-miR-92b-3p | 1.745054201 | mmu-miR-3963 | -1.51124185 |
| mmu-miR-871-3p | 1.726468026 | mmu-let-7i-5p | -1.51100143 |
| mmu-miR-132-5p | 1.698063757 |  |  |
| mmu-miR-5112 | 1.691938666 |  |  |
| mmu-miR-1933-3p | 1.674848376 |  |  |
| mmu-miR-301b-5p | 1.668398073 |  |  |
| mmu-miR-201-5p | 1.667898063 |  |  |
| mmu-miR-714 | 1.646719304 |  |  |
| mmu-miR-20b-3p | 1.620769817 |  |  |
| mmu-miR-465b-5p | 1.614851513 |  |  |
| mmu-miR-675-3p | 1.597942073 |  |  |
| mmu-miR-96-3p | 1.591419861 |  |  |
| mmu-miR-497a-5p | 1.580268981 |  |  |
| mmu-miR-743b-5p | 1.563120091 |  |  |
| mmu-miR-3070-5p | 1.560482221 |  |  |
| mmu-miR-344b-5p | 1.559618124 |  |  |
| mmu-miR-463-3p | 1.557677273 |  |  |
| mmu-miR-194-2-3p | 1.556687114 |  |  |
| mmu-miR-679-5p | 1.547782457 |  |  |
| mmu-miR-654-3p | 1.539357598 |  |  |
| mmu-miR-471-5p | 1.5293649 |  |  |
| mmu-miR-127-3p | 1.525854461 |  |  |
| mmu-miR-652-5p | 1.511354079 |  |  |
| mmu-miR-1964-5p | 1.500754649 |  |  |

**Table S4** Fold-changes for miRNAs expression in cardiac tissues of mice differently regulated by CR initiated in different ages (CR group vs. AL group).

| **mmu-miRNA** | **CR group VS AL group (fold changes)** | | |
| --- | --- | --- | --- |
|  | **Young (6m)** | **Middle-aged (15m)** | **Old (22m)** |
| **miRNAs increased in young mice but decreased or remain unchanged in middle-aged、old mice after CR** | | | |
| mmu-miR-2137 | 1.97 | 1.08 | -1.11 |
| mmu-miR-1892 | 1.8 | -1.32 | -1.69 |
| mmu-miR-1981-5p | 1.79 | -3.13 | 1.32 |
| mmu-miR-669d-2-3p | 1.77 | 1.22 | -1.29 |
| mmu-miR-877-5p | 1.73 | -4.35 | -2.86 |
| mmu-miR-503-5p | 1.59 | 1.11 | 1.02 |
| mmu-miR-1931 | 1.57 | -3.03 | -1.18 |
| **miRNAs decreased in young mice but increased or remain unchanged in middle-aged、old mice after CR** | | | |
| mmu-miR-146a-5p | -5.55 | 6.51 | 1.07 |
| mmu-miR-374b-5p/mmu-miR-374c-5p | -3.57 | 2.18 | -1.11 |
| mmu-miR-146b-5p | -3.57 | 4.22 | 1.08 |
| mmu-miR-463-3p | -3.03 | 1.91 | 1.56 |
| mmu-miR-3066-3p | -2.43 | 1.7 | -1.29 |
| mmu-miR-19a-3p | -2.08 | 1.68 | -1.06 |
| mmu-miR-743b-3p | -2.04 | 36.8 | 1.13 |
| mmu-miR-1a-1-5p | -2 | 1.93 | -1.29 |
| mmu-miR-497a-5p | -1.96 | 17.92 | 1.58 |
| mmu-miR-149-5p | -1.96 | 1.49 | -1.28 |
| mmu-miR-1839-3p | -1.96 | -1.13 | 1 |
| mmu-miR-199a-5p | -1.96 | 1.48 | -1.12 |
| mmu-miR-208a-5p | -1.92 | 1.55 | -1.23 |
| mmu-miR-208a-3p | -1.88 | 1.12 | -1.17 |
| mmu-miR-499-3p | -1.85 | 13.04 | 1.22 |
| mmu-miR-106a-5p | -1.81 | 2.15 | -1.17 |
| mmu-miR-5099 | -1.78 | 1.08 | -1.12 |
| mmu-miR-17-5p | -1.78 | 3.1 | -1.21 |
| mmu-miR-122-3p | -1.75 | 8.09 | 1.09 |
| mmu-let-7i-3p | -1.75 | 1.74 | -1.05 |
| mmu-miR-652-3p | -1.72 | 1.45 | 1.12 |
| mmu-let-7b-5p | -1.69 | 1.91 | -1.2 |
| mmu-miR-26a-5p | -1.69 | 1.04 | -1.28 |
| mmu-miR-499-5p | -1.69 | 2.84 | -1.11 |
| mmu-miR-27a-3p | -1.66 | 1.39 | 1.04 |
| mmu-miR-133a-5p | -1.66 | 1.42 | -1.19 |
| mmu-miR-152-3p | -1.66 | 3.13 | -1.05 |
| mmu-miR-23b-3p | -1.63 | 1.42 | -1.17 |
| mmu-miR-98-5p | -1.63 | 1.28 | 1.04 |
| mmu-miR-378a-5p | -1.63 | 1.32 | -1.05 |
| mmu-miR-186-5p | -1.63 | -1.42 | -1.03 |
| mmu-miR-15a-5p | -1.63 | 1.58 | -1.14 |
| mmu-miR-1a-2-5p | -1.61 | 1.82 | -1.31 |
| mmu-miR-196a-1-3p | -1.61 | 4.12 | -1.12 |
| mmu-miR-16-5p | -1.61 | 1.17 | -1.23 |
| mmu-miR-29c-3p | -1.61 | -1.2 | -1.09 |
| mmu-let-7g-5p | -1.58 | 1.5 | -1.19 |
| mmu-miR-30e-3p | -1.58 | 1.19 | -1.29 |
| mmu-miR-101a-3p/mmu-miR-101c | -1.58 | 1.38 | -1.13 |
| mmu-miR-193a-3p | -1.56 | 5.04 | 1.2 |
| mmu-miR-30e-5p | -1.56 | 1.05 | -1.36 |
| mmu-miR-99a-5p | -1.56 | -1.07 | -1.07 |
| mmu-miR-138-2-3p | -1.56 | 3.04 | 2.06 |
| mmu-miR-125a-5p | -1.56 | 1.08 | -1.2 |
| mmu-miR-127-3p | -1.56 | 3.92 | 1.53 |
| mmu-miR-191-5p | -1.56 | 1 | -1.09 |
| mmu-miR-26b-5p | -1.56 | 1.58 | -1.19 |
| mmu-miR-30b-5p | -1.53 | 1.14 | -1.49 |
| mmu-miR-29b-3p | -1.53 | 1.24 | 1 |
| mmu-miR-3070-5p | -1.53 | 1.49 | 1.56 |
| mmu-miR-344e-3p | -1.53 | 2.78 | 1.19 |
| mmu-miR-196a-2-3p | -1.53 | 3.08 | 1.06 |
| mmu-miR-148a-3p | -1.53 | 3.04 | -1.16 |
| mmu-miR-19b-3p | -1.53 | 1.53 | 1.05 |
| mmu-miR-27b-3p | -1.53 | 1.94 | -1.19 |
| mmu-miR-184-5p | -1.53 | -1.07 | -1.14 |
| mmu-miR-126a-5p | -1.53 | -1.11 | -1.19 |
| mmu-miR-24-2-5p | -1.51 | 1.59 | -1.03 |
| mmu-miR-195a-5p | -1.51 | 2.47 | -1.02 |
| mmu-miR-1264-3p | -1.51 | -1.36 | -1.31 |
| mmu-miR-23a-3p | -1.51 | 1.59 | -1.12 |
| mmu-miR-1983 | -1.51 | 1.21 | -1.2 |
| **miRNAs increased in middle-aged and old mice but decreased or remain unchanged in young mice after CR** | | | |
| mmu-miR-34b-5p | 1.24 | 65.9 | 5.66 |
| mmu-miR-201-5p | 1.27 | 22.32 | 1.67 |
| mmu-miR-497a-5p | -1.96 | 17.92 | 1.58 |
| mmu-miR-92b-3p | 1.29 | 11.79 | 1.75 |
| mmu-miR-34c-5p | 1.26 | 4.8 | 2.87 |
| mmu-miR-5112 | 1 | 4.17 | 1.69 |
| mmu-miR-127-3p | -1.56 | 3.92 | 1.53 |
| mmu-miR-138-2-3p | -1.56 | 3.04 | 2.06 |
| mmu-miR-679-5p | 1.09 | 3.01 | 1.55 |
| mmu-miR-291b-3p | -1.25 | 2.41 | 1.98 |
| mmu-miR-743a-3p | -1.35 | 1.97 | 2.07 |
| mmu-miR-463-3p | -3.03 | 1.91 | 1.56 |
| **miRNAs decreased in middle-aged and old mice but increased or remain unchanged in young mice after CR** | | | |
| mmu-miR-877-5p | 1.73 | -4.34 | -2.85 |
| mmu-miR-425-3p | -1.11 | -4.34 | -1.96 |
| mmu-miR-705 | 1.06 | -4 | -2.22 |
| mmu-miR-30b-3p | -1.19 | -2.56 | -1.81 |

**Table S5** Sequences of primers used.

| **mRNAS** |  | | **5’-3’** |
| --- | --- | --- | --- |
| ANF | forward | | GAGTCTGTAGTTGTCGCAGTTG |
|  | reverse | | GGCCTTTTTGGAAGGAAGAGGA |
| BNP | forward | | GAGGTCACTCCTATCCTCTGG |
|  | reverse | | GCCATTTCCTCCGACTTTTCTC |
| β -MHC | forward | | ACTGTCAACACTAAGAGGGTCA |
|  | reverse | | TTGGATGATTTGATCTTCCAGGG |
| TGFβ1 | forward | | CTCCCGTGGCTTCTAGTGC |
|  | reverse | | GCCTTAGTTTGGACAGGATCTG |
| MMP2 | forward | | CAAGTTCCCCGGCGATGTC |
|  | reverse | | TTCTGGTCAAGGTCACCTGTC |
| MMP9 | forward | | CTGGACAGCCAGACACTAAAG |
|  | reverse | | CTCGCGGCAAGTCTTCAGAG |
| Col1a1 | forward | | GCTCCTCTTAGGGGCCACT |
|  | reverse | | CCACGTCTCACCATTGGGG |
| Col3a1 | forward | | CTGTAACATGGAAACTGGGGAAA |
|  | reverse | | CCATAGCTGAACTGAAAACCACC |
| F4/80 | forward | | CTTTGGCTATGGGCTTCCAGTC |
|  | reverse | | GCAAGGAGGACAGAGTTTATCGTG |
| MCP1 | forward | | GGCTCAGCCAGATGCAGTTAA |
|  | reverse | | CCTACTCATTGGGATCATCTTGCT |
| TNF-α | forward | | CCCTCACACTCAGATCATCTTCT |
|  | reverse | | GCTACGACGTGGGCTACAG |
| IL-1β | forward | | GCAACTGTTCCTGAACTCAACT |
|  | reverse | | ATCTTTTGGGGTCCGTCAACT |
| IL-6 | forward | | CCAGAGATACAAAGAAATGATGG |
|  | reverse | | ACTCCAGAAGACCAGAGGAAAT |
| IL-10 | forward | | GCTCTTACTGACTGGCATGAG |
|  | reverse | | CGCAGCTCTAGGAGCATGTG |
| p16^ink4a^ | forward | | GAACTCTTTCGGTCGTACCC |
|  | reverse | | CGAATCTGCACCGTAGTTGA |
| p53 | forward | | TTCTGTAGCTTCAGTTCATTGG |
|  | reverse | | ATGGCAGTCATCCAGTCTTC |
| FOXO1 | forward | | CCCAGGCCGGAGTTTAACC |
|  | reverse | | GTTGCTCATAAAGTCGGTGCT |
| FOXO3 | forward | | CTGGGGGAACCTGTCCTATG |
|  | reverse | | TCATTCTGAACGCGCATGAAG |
| FOXO4 | forward | | CTTCCTCGACCAGACCTCG |
|  | reverse | | ACAGGATCGGTTCGGAGTGT |
| β-actin | forward | | TAAAGACCTCTATGCCAACACAGT |
|  | reverse | | CACGATGGAGGGGCCGGACTCATC |
| **miRNAs** | | **5’-3’** | |
| mmu-miR-2137 | sequence | | GCCGGCGGGAGCCCCAGGGAG |
|  | forward | | GCCGGCGGGAGCCCCAGGGAG |
| mmu-miR-1892 | sequence | | AUUUGGGGACGGGAGGGAGGAU |
|  | forward | | ATTTGGGGACGGGAGGGAGGAT |
| mmu-miR-146a-5p | sequence | | UGAGAACUGAAUUCCAUGGGUU |
|  | forward | | TGAGAACTGAATTCCATGGGTT |
| mmu-miR-146b-5p | sequence | | UGAGAACUGAAUUCCAUAGGCU |
|  | forward | | TGAGAACTGAATTCCATAGGCT |
| mmu-miR-19a-3p | sequence | | UGUGCAAAUCUAUGCAAAACUGA |
|  | forward | | TGTGCAAATCTATGCAAAACTGA |
| mmu-miR-34b-5p | sequence | | AGGCAGUGUAAUUAGCUGAUUGU |
|  | forward | | AGGCAGTGTAATTAGCTGATTGT |
| mmu-miR-34c-5p | sequence | | AGGCAGUGUAGUUAGCUGAUUGC |
|  | forward | | AGGCAGTGTAGTTAGCTGATTGC |
| mmu-miR-201-5p | sequence | | UACUCAGUAAGGCAUUGUUCUU |
|  | forward | | TACTCAGTAAGGCATTGTTCTT |
| mmu-miR-138-2-3p | sequence | | GCUAUUUCACGACACCAGGGU |
|  | forward | | GCTATTTCACGACACCAGGGT |
| Rnu6 | sequence | | GTGCTCGCTTCGGCAGCACATATACTAAAATTGGAACGATACAGAGAAGATTAGCATGGCCCCTGCGCAAGGATGACACGCAAATTCGTGAAGCGTTCCATATTTT |
|  |  |  |  |
|  |  |  |  |
|  | forward | | CTCGCTTCGGCAGCACATA |
| universal downstream primer | | | GCGAGCACAGAATTAATACGAC |

**Table S6** NRC micronutrient recommendations for mice versus the micronutrient contents provided by diet we used in 40% CR. 1NRC recommendation for mice (National Research Council, 1995).

| **Micronutrients** | **Growth recommendations^1^** | **Our diet** | |
| --- | --- | --- | --- |
|  | **(unit/100 g mouse)** | **unit/100g** | **unit/60g** |
| **Minerals** | | | |
| Calcium (g) | 0.50 | 1.22 | 0.732 |
| Phosphorus (g) | 0.30 | 0.86 | 0.516 |
| Magnesium (g) | 0.05 | 0.18 | 0.108 |
| Potassium (g) | 0.05 | 0.59 | 0.354 |
| Sodium (g) | 0.05 | 0.22 | 0.132 |
| Copper (mg) | 0.50 | 2.90 | 1.74 |
| Iron (mg) | 3.50 | 40.00 | 24 |
| Manganese (mg) | 1.00 | 6.10 | 3.66 |
| Zinc (mg) | 1.50 | 14.00 | 8.4 |
| Selenium (ug) | 15.00 | 54.00 | 32.4 |
| Iodine (ug) | 15.00 | 282.00 | 169.2 |
| **Vitamins** | | | |
| Cholecalciferol (ug) | 2.00 | 12.6 | 7.56 |
| dl-α-Tocopheryl acetate (mg) | 1.80 | 17.70 | 10.62 |
| Biotin (mg) | 0.02 | 0.052 | 0.0312 |
| Choline (mg) | 75.00 | 184.00 | 110.4 |
| Folic acid (mg) | 0.10 | 0.544 | 0.3264 |
| Niacin (mg) | 1.50 | 11.67 | 7.002 |
| Pantothenic acid (mg) | 1.00 | 3.94 | 2.364 |
| Riboflavin (mg) | 0.30 | 1.32 | 0.792 |
| Thiamin (mg) | 0.40 | 6.96 | 4.176 |
| Pyridoxine (mg) | 0.60 | 1.98 | 1.188 |
| Cobalamin (ug) | 5.00 | 7.00 | 4.2 |

**National Research Council (NRC).**

**Method S1** DNA isolation and measurement of telomere length by RT-qPCR.

Genomic DNA was extracted from the heart tissue using a Genomic DNA Isolation Kit (Invitrogen). The relative telomere length was measured using RT-qPCR as described previously ([Callicott & Womack 2006](#_ENREF_6)). The assay involved comparing the abundance of telomere DNA to the single copy genomic DNA number for each sample and by further comparison of normalized value between DNAs of different sources. The ratio of the telomere (T) and single-copy *36B4* gene (S) reflect the length of telomeres (T/S ratio=2^-[Ct (telo)-Ct (36B4)]^=2^-∆Ct^). The relative T/S ratio (T/S of one sample relative to another sample) is 2^-(∆Ct1-∆Ct2)^=2^-∆∆Ct^. The primer sequences were as follows: telomere: forward, 5'-CGGTTTGTTTGGGTTTGGGTTTGGGTTTGGGTTTGGGTT-3' and reverse, 5'-GGCTTGCCTTACCCTTACCCTTACCCTTACCCTTACCCT-3'; 36B4: forward, 5'-ACTGGTCTAGGACCCGAGAAG-3' and reverse, 5'-TCAATGGTGCCTCTGGAGATT-3'. To measure the telomere length, triplicate 25µL PCR reactions included 12.5µL SYBR Green PCR Master Mix (Roche), 900nM forward and reverse primers for telomere, whereas 400nM forward and reverse primers for 36B4, 20ng genomic DNA, and sufficient double-distilled H_2_O. The PCR conditions were as follows: 10min at 95°C, followed by 40 cycles of 15 s at 95°C, and 60 s at 60°C. The temperature was heated from 60°C to 95°C, and the PCR melting curve was obtained every 1.0°C after the amplification reaction.

**Method S2** Microarray analysis

The ventricular tissue samples used for mRNA and miRNA array analysis were obtained from young, middle-aged, and old mice with or without CR (n=6/group). A pool containing equivalent amounts of mixed ventricular tissues for each group was prepared. A total of 6 RNA samples representing 6 individual groups were isolated using Trizol and purified with the RNeasy Mini Kit (Qiagen, Valencia, CA, USA) according to the manufacturer’s instructions. The RNA quality and quantity was measured by Nanodrop spectrophotometer (ND-1000, Nanodrop Technologies, Wilmington, DE, USA). For miRNA array, total RNA (1µg) from each group was labeled using the miRCURY™ Hy3™/Hy5™ Power Labeling Kit (Exiqon, Vedbaek, Denmark) and hybridized with the miRCURY^TM^ LNA Array (v.18.0) (Exiqon), according to the array manual. Then, the slides were scanned using the Axon GenePix 4000B microarray scanner (Axon Instruments, Foster City, CA, USA). The scanned images were then imported into GenePix Pro 6.0 software (Axon) for grid alignment and data extraction. For mRNA array, total RNA (1µg) from each group was linearly amplified and labeled with Cy3-UTP. The labeled cRNAs were then hybridized to Agilent Whole Mouse Genome Microarray (Agilent, 4×44 K) using an Agilent Gene Expression Hybridization Kit (Agilent p/n 5188-5242) as recommended by the manufacturer. Consecutive to microarray washing, the processed slides were scanned with an Agilent DNA microarray scanner (Agilent p/n G2565BA). The raw gene expression data were extracted from Agilent Feature Extraction Software (version 11.0.1.1) and imported into Agilent GeneSpring GX software (version12.1) for further analysis. The microarray data have been deposited in the NCBI Gene Expression Omnibus (GEO) database (http://www.ncbi.nlm.nih.gov/geo/) under accession no. GSE89909.

The differentially expressed (DE) genes and miRNA were identified using a threshold of fold-change ≥2 and 1.5, respectively. Pathway analysis was performed using the DAVID tool to map DE genes to Kyoto Encyclopedia of Genes and Genomes (KEGG). Two prediction databases, TargetScan and miRDB, were used for putative target gene prediction.

**Method S3** Transmission electron microscopy

Rod-shaped pieces of ventricle myocardium were fixed with a mixture of 2.5% glutaraldehyde, 1.25% paraformaldehyde, and 0.03% picric acid in 0.1M sodium cacodylate buffer (pH 7.4) overnight at 4°C. Subsequently, the tissue was washed in 0.1M cacodylate buffer, fixed with 1% osmium tetroxide/1.5% potassium ferrocyanide for 1h, washed with water, and stained with 1% aqueous uranyl acetate for 30 min followed by dehydration by alcohol gradient (5 min in 70%, 5 min in 90%, and 2, 3, and 5 min each in 100%). The samples were then infiltrated and embedded in TAAB Epon (Marivac Canada, Inc., St. Laurent, QC, Canada). The ultrathin sections (60 nm) were cut on a Reichert Ultracut-S microtome, placed onto copper grids, stained with uranyl acetate and lead citrate, and examined with a Tecnai G2 Spirit BioTWIN transmission electron microscope (FEI, Hillsboro, USA). Mitochondria (total and damaged) and lipid droplets were quantified blindly in 20 images (2800× magnification) from 3 hearts in each group.

Damaged mitochondria were quantified blindly in 20 images from 3 hearts in each group (2800× magnification) using measuring module of OLYMPUS cellSens Dimension (Olympus, Tokyo, Japan). Damaged mitochondria were defined by vacuolation (loss of matrix density) or reduced numbers of cristae in more than 20% of the area of a mitochondrion.

**Method S4** Histopathological analysis

The ventricular tissues were fixed in 4% paraformaldehyde solution, embedded in paraffin, sectioned (3μm), and then stained with the fluorescein isothiocyanate (FITC)-conjugated WGA (Sigma, St. Louis, MO, USA). The cardiomyocyte cross-sectional area was quantitated from 200 randomly selected cardiomyocytes at a magnification of 400× by fluorescence microscopy (Olympus, Tokyo, Japan). The myocardial fibrotic area was assessed using Masson’s trichrome staining (Sigma). The ventricular tissues were incubated with primary antibody against LC3II (Proteintech Group, Chicago, IL, USA) at 4°C overnight, followed by the FITC-conjugated secondary antibody (Jackson ImmunoResearch Inc, West Grove, PA, USA) for 60 min at room temperature. Hoechst 33342 (Invitrogen, Carlsbad, CA, USA) was used to counterstain the nuclei. The number of LC3II dots was counted and analyzed in 15 randomly selected images (400× magnification) from 3 hearts in each group.

**Method S5** RT-qPCR of mRNA and miRNA

Total RNA was extracted from ventricular tissues using Trizol reagent (Invitrogen) according to the manufacturer’s instructions. Total RNA (2μg) was reverse-transcribed to cDNA. RT-qPCR was performed with SYBR Green PCR Master Mix (Roche, Branchburg, NJ, USA) on an ABI Prism 7000 sequence detection system (Applied Biosystems, Foster City, CA, USA). Each sample was analyzed in triplicate, and relative gene expression was normalized to β-actin. The PCR primers were designed by Primer5 software (Table S5).

For miRNA, poly (A) tailing and cDNA synthesis were performed using the RevertAid RT Reverse Transcription Kit (Thermo Scientific, Waltham, [MA](https://www.baidu.com/s?wd=MA&tn=44039180_cpr&fenlei=mv6quAkxTZn0IZRqIHckPjm4nH00T1YkPhcsPjmznHndPhwhPhP-0ZwV5Hcvrjm3rH6sPfKWUMw85HfYnjn4nH6sgvPsT6KdThsqpZwYTjCEQLGCpyw9Uz4Bmy-bIi4WUvYETgN-TLwGUv3EPjRvnHmdrHm1P1bkPj0Lnj6d), USA). Next, the reverse-transcribed product was amplified and monitored in RT-qPCR using a miRNA-specific upstream primer and the universal downstream primer (Table S5). The expression levels were normalized to that of small noncoding RNA U6 (Rnu6).

**Method S6** Western blot analysis

Total protein was extracted using a RIPA lysis buffer and Western blotted using antibodies against p16, p53, TGFβ1, Gapdh (Bioworld Technology Inc., Louis Park, MN, USA), col3a1 (Proteintech), col1a1, F4/80 (Santa Cruz Biotechnology, Santa Cruz, CA, USA), α-tubulin, LC3A/B, Beclin-1, p62, Phospho-AMPKα (Thr172), and AMPKα (Cell Signaling Technology, Beverly, MA, USA).
